# Synthesis of novel coumarin nucleus-based DPA drug-like molecular entity: *In vitro* DNA/Cu(II) binding, DNA cleavage and pro-oxidant mechanism for anticancer action

**DOI:** 10.1371/journal.pone.0181783

**Published:** 2017-08-01

**Authors:** Saman Khan, Ali Mohammed Malla, Atif Zafar, Imrana Naseem

**Affiliations:** 1 Department of Biochemistry, Faculty of Life Sciences, Aligarh Muslim University, Aligarh, Uttar Pradesh, India; 2 Department of Chemistry, Government Degree College, Sopore, Kashmir, India; Universite du Quebec a Trois-Rivieres, CANADA

## Abstract

Despite substantial research on cancer therapeutics, systemic toxicity and drug-resistance limits the clinical application of many drugs like cisplatin. Therefore, new chemotherapeutic strategies against different malignancies are needed. Targeted cancer therapy is a new paradigm for cancer therapeutics which targets pathways or chemical entities specific to cancer cells than normal ones. Unlike normal cells, cancer cells contain elevated copper which plays an integral role in angiogenesis. Copper is an important metal ion associated with chromatin DNA, particularly with guanine. Thus, targeting copper via copper-specific chelators in cancer cells can serve as an effective anticancer strategy. New pharmacophore di(2-picolyl)amine (DPA)-3(bromoacetyl) coumarin (ligand-L) was synthesized and characterized by IR, ESI-MS, ^1^H- and ^13^C-NMR. Binding ability of ligand-L to DNA/Cu(II) was evaluated using a plethora of biophysical techniques which revealed ligand-L-DNA and ligand-L-Cu(II) interaction. Competitive displacement assay and docking confirmed non-intercalative binding mode of ligand-L with ctDNA. Cyclic voltammetry confirmed ligand-L causes quasi reversible Cu(II)/Cu(I) conversion. Further, acute toxicity studies revealed no toxic effects of ligand-L on mice. To evaluate the chemotherapeutic potential and anticancer mechanism of ligand-L, DNA damage via pBR322 cleavage assay and reactive oxygen species (ROS) generation were studied. Results demonstrate that ligand-L causes DNA cleavage involving ROS generation in the presence of Cu(II). In conclusion, ligand-L causes redox cycling of Cu(II) to generate ROS which leads to oxidative DNA damage and pro-oxidant cancer cell death. These findings will establish ligand-L as a lead molecule to synthesize new molecules with better copper chelating and pro-oxidant properties against different malignancies.

## Introduction

DNA is the genetic material which regulates vital processes such as transcription, recombination, proliferation and cell survival. Targeting DNA to modify or inhibit its activity is a subject of extensive research for designing new anticancer agents against different malignancies [1]. Among various anticancer agents, DNA cleaving agents have attracted considerable interest in the field of molecular biology and drug development [2]. Metal-containing compounds have been developed with extensive applications in wide ranging fields such as material and biological sciences [3, 4]. The present treatment regiments for chemotherapy under the class of metal-containing compounds such as platinum-based drug (cisplatin) for different malignancies. However, the major disadvantages of cisplatin (heavy-metal based drug) include various side effects such as severe systemic toxicity (hepatotxicity and nephrotoxicity) and increased drug resistance [5, 6].

To minimize the toxicity and side effects of metal-containing drugs, new chemotherapeutic agents and therapies against different malignancies need to be developed. Targeted cancer therapy is a relatively new form of therapy which involves the use of drugs that block the growth of cancerous tissue by interfering with specific molecules/pathways and spare the normal cells [7]. The basic rationale of targeted cancer therapy is to target chemical entities/mutated proteins that are specific to cancer cells and absent in normal cells of the body [8].

One major difference between normal and cancer cells is the levels of copper which are elevated in malignant cells [9–11]. Copper is an important redox active metal ion attached to DNA bases, particularly guanine [12]. Elevated copper in cancer plays a role in angiogenesis by functioning as a co-factor of several pro-angiogenic molecules such as vascular endothelial growth factor (VEGF), angiogenin and basic fibroblast growth factor (bFGF) [13,14]. Copper chelating to decrease copper bioavailability has been investigated in many clinical studies to inhibit angiogenesis (e.g. clinicaltrials.gov id# NCT00003751, NCT00176800, NCT01837329, NCT02068079, NCT00405574) [15]. Many copper chelators have been synthesized to possess anticancer activities. For example, in murine model of hepatocellular carcinoma, trientine induced apoptosis through interaction with cellular copper and ROS generation [16]. Lowering of copper by tetrathiomolybdate has been observed to reduce tumor growth and impede angiogenesis in murine mesothelioma tumor model [17]. D-penicillamine also shows anticancer activity against leukemia and breast cancer by chelating intracellular bioavailable copper [18]. Thus, under targeted cancer therapy copper-specific chelators should to be synthesized to lower bio-available copper and provide selective approach to target tumor cells. Cytotoxicity of copper-specific chelators arises from high redox activity with copper ions to produce reactive oxygen species (ROS) which leads to DNA damage and oxidative cell death [19, 20].

Coumarins (2*H*-1-benzopyran-2-one; 1,2-benzopyrone; *cis-o*-coumarinic acid lactone; coumarinic anhydride) are a large group of naturally occurring organic compounds synthesized by plants, bacteria and fungi [21, 22]. Coumarin represents a promising scaffold and used for the synthesis of coumarin-based derivatives which exhibit various pharmacological properties such as antifungal, antioxidant, anticoagulant, antiviral, antiproliferative, antialzheimer, anticancer and anti-HIV [23–30]. The biochemical properties of coumarin and its derivatives have suggested their use in clinical medicine [31]. Various coumarin derivatives are under clinical trials in different malignancies such as prostate cancer, renal cell carcinoma, malignant melanoma, breast cancer and leukemia [32, 33]. Recently, several studies were conducted that described the addition of bioactive moieties such as pyran, pyridine, thiazole and pyrazole into coumarin nucleus to synthesize new anticancer agents [34–37]. However, no progress has been made towards efficient synthesis of coumarin-based dipic derivatives. Dipic ligands such as di(2-picolyl)amine (DPA) and N-substituted DPA derivatives have been investigated for anticancer activities against malignant cells [38, 39]. Also, it is important to note that DPA exhibits co-ordination properties in metal complexes [40].

In continuation of our pursuit to design and synthesize copper-specific chelators against cancer cells, we herein report the synthesis, characterization and biological activity of new coumarin nucleus-based DPA derivative (ligand-L). Binding studies of ligand-L to DNA/Cu(II) were explored to ascertain the interaction and binding mode. pBR322 DNA cleavage experiments were also conducted to assess the DNA damage caused by ligand-L induced ROS generation in the presence of Cu(II). Results indicate that ligand-L acts as pro-oxidant in the presence of Cu(II) leading to its cytotoxic action. Ligand-L also follows all the parameters under ‘Rule of five’ (no violations) showing tendency towards drug-likeness and drug score with no toxicity. The results of this investigation would be useful in establishing ligand-L as a lead molecule to synthesize new chemical molecules with better copper chelating and pro-oxidant properties against cancer cells.

## Materials and methods

### General experimental procedures

Chemicals and solvents used in this study were purchased from Merck, India and Sigma-Aldrich, St. Louis, MO, USA and used without further purification. IR spectrum was recorded using Perkin Elmer Spectrum Two IR spectrometer by potassium bromide (KBr) pellet method and values are given in cm^-1^. ^1^H and ^13^C NMR spectra were run in DMSO-*d*_*6*_ on a Bruker Avance II 400 NMR spectrometer at 400 and 100 MHz, respectively. Chemical shifts are reported in ppm (Δ) relative to internal standard tetramethylsilane (TMS). Mass spectrum was recorded on a JEOL SX 102/DA-6000 mass spectrometer. Melting point was recorded on Buchi melting point apparatus B-545. Elemental analysis (C, H, N) was recorded on Perkin Elmer 2400 Series II system. Thin layer chromatography (TLC) glass plates (20×5 cm) were coated with silica gel G and exposed to iodine vapours to check the homogeneity as well as progress of the reaction.

### General method for the synthesis of coumarin-based di(2-picolyl)amine (DPA) derivative

An equimolar mixture of 3-(2-bromoacetyl)-2*H*-chromen-2-one and di(2-picolyl)amine (DPA) (2 mmol each), was stirred in dichloromethane (20 ml) in the presence of sodium carbonate. The reaction mixture was allowed to stir at room temperature for 6–8 h. After completion of the reaction, as evident from TLC, the solvent was removed under reduced pressure. The crude product obtained was washed with water, dried and crystallized from appropriate solvents.

### 3-(2-{bis[(pyridin-2-yl)methyl]amino}acetyl)-*2H*-chromen-2-one (ligand-L)

Ligand-L crystallized from CHCl_3_-MeOH as light reddish solid; Yield: 89%; mp 203°C; IR (KBr, cm^-1^): 1731.25 (lactone carbonyl-*coumarin nucleus*), 1688.87 (*α*, *β*-unsaturated carbonyl), 1613.28 (*α*, *β*- C = C). ^1^H NMR (400 MHz, DMSO-*d*_*6*_, Δ, ppm): 3.82 (s, 4H), 4.15 (s, 2H), 7.20–7.53 (m, 8H), 7.64–7.68 (m, 2H), 7.81 (d, 2H), 8.51 (s, 1H). ^13^C NMR (100 MHz, DMSO-*d*_*6*_, Δ, ppm): 61.34, 61.35, 64.20, 116.53, 121.80, 121.83, 124.20, 124.23, 125.81, 127.90, 128.62, 131.24, 136.31, 139.25, 139.29, 149.45, 149.48, 152.20, 153.09, 157.82, 157.85, 166.81, 181.79. Electrospray ionization mass spectrometry (ESI-MS): m/z 385 [M^+^]. Anal. Calcd. for C_23_H_19_N_3_O_3_: C, 71.67; H, 4.97; N, 10.90. Found: C, 71.64; H, 4.98; N, 10.91.

### Calf thymus DNA (ctDNA) preparation: DNA binding experiments

Stock solution of ctDNA was prepared in 10 mM Tris-HCl buffer (pH 7.2) and later stored at 4°C. Purity of DNA solution was analyzed by recording the absorbance ratio i.e. A_260nm_ / A_280nm_. Absorbance ratio was between 1.8 and 1.9, and therefore no further purification was required. DNA concentrations used in different experiments were determined using average molar extinction coefficient value of 6600 M^-1^ cm^-1^ of a single nucleotide at 260 nm [41]. Stock solution (3 mM) of ligand-L was prepared in DMSO.

### UV-Vis spectroscopy of ligand-L with ct-DNA

UV-Vis spectral study of ligand-L was carried out using UV-VIS spectrophotometer (UV-1800, Shimadzu Corp., Tokyo, Japan). Absorbance spectra of ligand-L were recorded in the absence and presence of increasing concentrations of ct-DNA. Briefly, fixed concentration of ligand-L (5 μM) was titrated with increasing concentrations of ct-DNA (0–35 μM) in 10 mM Tris-HCl buffer (pH 7.2).

### UV-Vis spectroscopy of ligand-L with Cu(II)

Absorption spectra of ligand-L in absence and presence of Cu(II) were recorded in the wavelength range 235–400 nm using UV-VIS spectrophotometer (UV-1800, Shimadzu Corp., Tokyo, Japan). To the reaction mixture, fixed concentration of ligand-L (5 μM) was used and titrated against increasing concentrations of Cu(II) (0–35 μM) in 10 mM Tris-HCl (pH 7.2).

### Steady state fluorescence studies of ligand-L with Cu(II) and ct-DNA

Fluorescence emission spectra were recorded on a RF-5301PC spectrofluorometer (Shimadzu Corp., Tokyo, Japan). Ligand-L was excited at 292 nm and emission spectra were recorded in the wavelength range 300–420 nm after setting the widths of excitation slit at 5 nm and emission slit at 10 nm. To a 1 ml reaction mixture, fixed concentration of ligand-L (5 μM) was used and titrated with increasing concentrations of ct-DNA/Cu(II) (0–40 μM). All fluorescence spectroscopy experiments were carried out in 10 mM Tris-HCl (pH 7.2). The fluorescence intensities were also corrected for inner filter effects using the equation [42]:

$$F_{corr}=F_{obs}10^{(A_{1}+A_{2})/2}$$

where, F_corr_ and F_obs_ are the corrected and observed fluorescence intensities, respectively and A_1_ and A_2_ are the sum of absorbances of DNA and ligand-L at the excitation (292 nm) and emission (350 nm) wavelengths, respectively.

### Competitive displacement assays

DNA binding dyes such as ethidium bromide (EtBr) [43] and Hoechst 33258 (HO) are used to decipher the binding mode of drug on interaction with DNA. In case of EtBr displacement assay, ctDNA (20 μM) and EtBr (2.5 μM) were dissolved in 10 mM Tris-HCl (pH 7.2). Later, increasing concentrations of ligand-L (0–50 μM) were added to EtBr-DNA solution. Solution was excited at 475 nm and emission spectra were recorded in the range 500–700 nm. Groove binding dye, HO (2 μg/ml) and ctDNA (20 μM) were dissolved in 10 mM Tris-HCl (pH 7.2) and titrated with increasing concentrations of ligand-L (0–50 μM). HO-DNA complex was excited at 343 nm and emission was recorded from 400–600 nm.

### Isothermal titration calorimetry measurements (ITC)

ITC is an informative and sensitive method to study thermodynamic parameters of interactions between bio-macromolecules and ligands [44]. Thermodynamic parameters resulted from interaction between ligand-L and ctDNA were determined using VP-ITC titration microcalorimeter (MicroCal Inc., Northampton, MA, USA). All solutions used for ITC were properly degassed prior to use. Briefly, reference and calorimeter cell were loaded with 1X TE buffer (pH 8.0) and ctDNA (0.8 mM), respectively. ctDNA was titrated with 4.1 mM ligand-L up to 28 successive injections of 10 μl each. Titration cell was stirred continuously at 307 rpm and reference power was set at 16 μcal sec^-1^. Calorimetric data was fitted using independent binding model and analyzed using MicroCal Origin 7.0 software to calculate equilibrium binding constant (K_b_), entropy change (ΔS^0^) and enthalpy change (ΔH^0^) of complex formation. Free energy change (ΔG^0^) of ligand-L-DNA complex was calculated using following equations:

$${\Delta G}^{0}=-RT\;ln\;K_{b}$$ (1)

$${\Delta G}^{0}={\Delta H}^{0}-T{\Delta S}^{0}$$ (2)

where, T is the absolute temperature (298 K) and R is the gas constant with value 8.314 J mol^-1^ K^-1^.

### Circular Dichroism (CD) studies

CD spectra of ctDNA and in the presence of increasing concentrations of ligand-L were recorded using JASCO-J-720 CD spectropolarimeter equipped with a Peltier-type temperature controller. CD experiments were carried out at 25°C. All the CD spectra were recorded in the range 228–300 nm with a scan speed 200 nm min^-1^ and spectral bandwidth of 1.0 nm. Each spectrum was the average of three scans. Background spectrum of buffer (10 mM Tris-HCl, pH 7.2) was subtracted from the spectra of DNA and ligand-L-DNA solution. The results were expressed as ellipticity (mdeg).

### Potassium iodide (KI) quenching studies

Iodide quenching experiments were performed in the presence and absence of ctDNA. Briefly, ligand-L (30 μM) was dissolved in 10 mM Tris-HCl (pH 7.2) and titrated with increasing concentrations of KI (0–8 mM). Ligand-L was excited at 292 nm and emission spectra were recorded in the wavelength range 300–420 nm. In a different experiment, ligand-L (30 μM) and ctDNA (30 μM) were taken and then an increasing concentration of KI (0–8 mM) was added. Quenching constant (K_sv_) values in the presence and absence of DNA was calculated via Stern-Volmer equation [45, 46].

### Molecular docking studies

Binding mode of ligand-L in B-DNA was determined using AutoDock (v4.2) in Lamarckian Genetic Algorithm [47, 48]. Ligand-L chemical structure was prepared using ChemDraw 12.0 and saved in MOL format. Mol file was converted into PDB using Avogadro 1.0.1 [49]. Later, structure optimization was carried out using AM1 (Austin Model 1) in Arguslab 4.0.1 and the best conformer exhibiting lowest energy was saved in PDB format for docking. Target receptor (PDBID: 1BNA, sequence d(CGCGAATTCGCG)_2_) and ligand-L were prepared via docking protocol and saved into ‘PDBQT’ format. Blind docking was then performed to determine the most favourable binding mode of ligand-L in DNA. The input ‘grid parameter’ files were adjusted to X = 60, Y = 60 and Z = 110 with 0.375 nm grid spacing. Rest all docking parameters were set to default values. Energy-scoring function is used to determine the top ligand-DNA pose. The top pose conformation was visualised via PyMOL software (Molecular Graphics System, version 1.5.0.1, Schrodinger.LLC) [50].

### Electrochemical measurements of Cu(II)/Cu(I) conversion in presence of ligand-L

Cyclic voltammetry studies were performed on Princeton Applied Research model 263A-1 potentiostat/galvanostat. Voltammetric experiments were carried out using a 3-electrode setup cell consisting of a glassy carbon disk as the working electrode, a platinum wire as the auxiliary electrode and Ag/AgCl electrode system saturated with potassium chloride (KCl) as the reference electrode. Experiments were performed with ligand-L solution in the absence and presence of Cu(II) ions. A solution of 10 mM Tris-HCl (pH 7.2) was used as a supporting electrolyte. Solutions were purged for 5 min before recording the voltammograms. Data was recorded at a scan rate of 150 mV sec^-1^ at 25°C.

### Prediction of drug-likeness (Lipinski’s rule of five)

Lipinski’s rule of five [51] is a rule of thumb that evaluates the drug-likeness properties of chemical compounds. To determine the drug-likeness of ligand-L, physiochemical properties such as octanol-water partition coefficient (log *P)*, molecular weight (MW), rotatable bonds, polar surface area, hydrogen bond donors and acceptors were calculated using molinspiration server (www.molinspiration.com/cgi-bin/properties) [52] and ChemAxon (www.chemicalize.org) [53].

### *In vitro* toxicity test for synthesized compound

*In vitro* toxicity of ligand-L was checked using erythrocyte lysis test. Briefly, fresh heparinised blood was collected from a healthy non smoking volunteer (Author herself) in EDTA tubes and then centrifuged at 1500 × g for 15 min at 4°C. After centrifugation, buffy coat and plasma present at the top was discarded and erythrocytes at the bottom of tube were washed three times with phosphate buffer saline (PBS) (pH 7.4). The washed erythrocytes were diluted in isotonic solution and 5% hematocrit was prepared for testing toxicity. Red blood cells (RBCs) suspension was incubated with 0.5 ml of 2% DMSO solution (vehicle control for ligand-L) and increasing concentrations of ligand-L (25–100 μM) at 37°C for 1 hr. After complete incubation, the reaction mixture was centrifuged at 1500 × g and the supernatant was collected to measure released (hemoglobin) Hb at λ_max_ = 576 nm.

Percent hemolysis was measured via the formula:

$$\frac{\left(A_{sample}-A_{control}\right)}{\left(A_{positive\;control}-A_{control}\right)}\times 100$$

where, A_sample_ is the absorbance of sample treated with ligand-L, A_control_ is the absorbance of samples incubated in PBS and A_positive control_ is the absorbance of samples treated with 1% Triton X-100.

### *In vivo* acute toxicity study

Ligand-L toxicity was assessed using organisation for economic co-operation and development (OECD) protocol [54]. All animal experiments were approved by Institutional Animal Ethical Committee (IAEC) of Department of Biochemistry, Faculty of Life Sciences, Aligarh Muslim University, Aligarh, India (714/02/a/CPCSEA). Twelve adult swiss albino mice (43–45 g) were maintained in hygienic large cages at 25 ± 2°C on a 12 h light/dark cycles and acclimatized for 1 week before the treatment. Mice were divided into two groups: (1) Group A (n = 6) received only vehicle control (DMSO) intraperitoneally; Group B (n = 6) received 250 mg/kg ligand-L dissolved in DMSO intraperitoneally. Animals were observed for 4 hours post injection and three times a day thereafter injection to notice any change in behaviour and physiological activities or mortality. After 14 days, the animals were sacrificed by cervical dislocation. Blood samples were collected, allowed to clot and centrifuged at 1000 × g for 15 min at room temperature for serum biochemical examination (liver and kidney function test). Kidney and liver were excised and processed for histological analysis via H&E staining.

### ROS measurement

ROS namely superoxide anion and hydroxyl radical production were assessed. Superoxide generation by ligand-L alone and in the presence of Cu(II) ions was determined using nitroblue tetrazolium (NBT) assay [55]. Briefly, the assay mixture contains 50 mM sodium phosphate buffer (pH 7.5), 0.3 mM NBT, 0.1 mM EDTA and 0.06% Triton X-100 in a total reaction volume of 3 ml. The reaction was started by adding ligand-L in the presence and absence of Cu(II) ions and absorbance was recorded at 560 nm against a blank solution after 1 h incubation. Hydroxyl radical production by ligand-L in the presence and absence of Cu(II) ions was detected by the method of Quinlan and Gutteridge [56]. ctDNA (300 μg) was used as a substrate and the generation of malondialdehyde from deoxyribose radicals was assayed by recording the absorbance at 532 nm.

### Plasmid nicking assay

DNA damage by Cu(II)-ligand-L interaction was assessed by plasmid nicking assay. Reaction mixture (25 μl) contained 10 mM Tris-HCl (pH 7.2), 0.5 μg pBR322 plasmid DNA and other components as indicated in legends. Incubation was performed for 1 h at 37°C. After complete incubation, 5 μl of 5X tracking dye (40 mM EDTA, 0.05% bromophenol blue and 50% (v/v) glycerol) was added and the complete reaction mixture was subjected to electrophoresis in 1% agarose gel. DNA bands were stained with 0.5 μg/ml EtBr solution and visualized under UV illumination gel-doc system (Bio-Rad; Hercules, CA).

### Statistical analysis

Experimental values were expressed as mean ± SEM of three independent experiments. Data was analysed by one way-analysis of variance (ANOVA) using GraphPad Prism 5.01 (California, USA) to examine statistically significant differences. p-values < 0.05 were considered statistically significant.

## Results

### Chemistry

**Fig 1:**
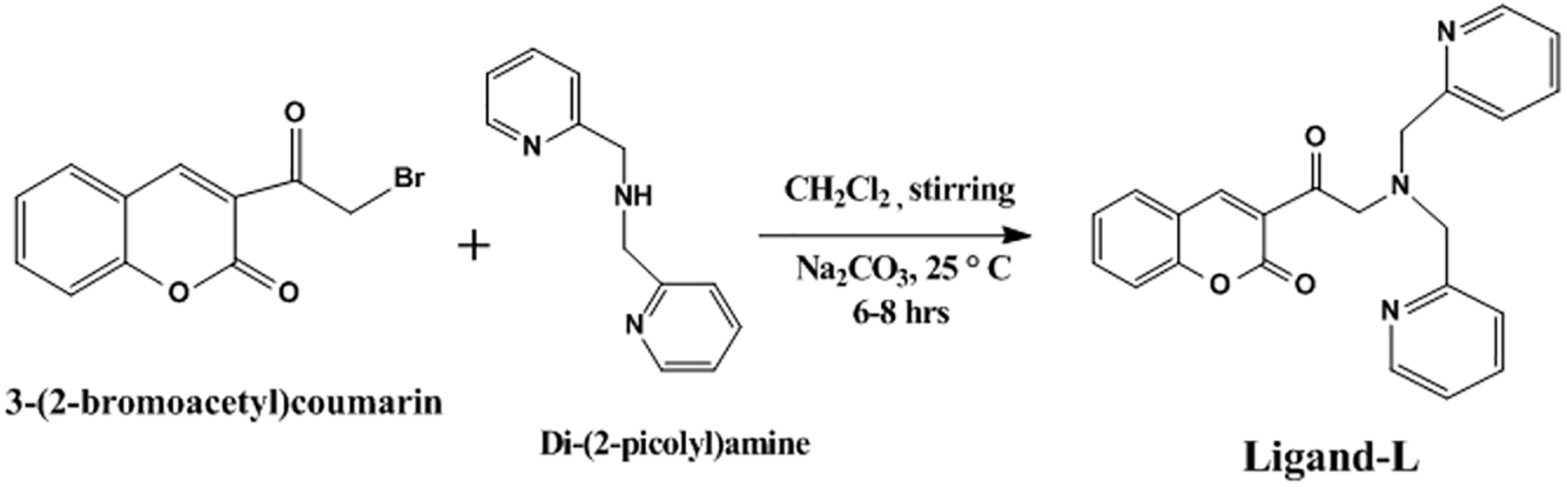
Scheme 1: Synthetic route for the synthesis of ligand-L.

Ligand-L was typically synthesized *via* a condensation reaction between 3-(2-bromoacetyl)-2*H*-chromen-2-one and di(2-picolyl)amine (DPA) with the elimination of HBr molecule under stirring at room temperature (Fig 1**)**. The compound was obtained in excellent yield (89%) with high degree of purity. The structure of ligand-L was characterized by IR, ^1^H NMR, ^13^C NMR, ESI-MS and elemental analysis. The characterisation studies have been in good corroboration with the expected structural framework of ligand-L. IR spectrum of ligand-L displayed characteristic signals for lactone carbonyl (coumarin nucleus) (1731.25 cm^-1^), *α*, *β*-unsaturated carbonyl (1688.87 cm^-1^) and *α*, *β*- C = C (1613.28 cm^-1^) (S1 Fig). In ^1^H NMR spectral analysis, ligand-L exhibited a sharp downfield singlet resonating at around Δ 8.51 ppm assigned to olefinic proton (H-4) (= C-H) (S2 Fig). This appreciable downfield shift of H-4 olefinic proton may be due to possible H-bonding with the adjacent carbonyl group. A pair of singlet peaks resonating at Δ 3.82 [-N-(CH_2_)_2_] and Δ 4.15 (-CO-CH_2_-N-) correspond to four and two methylene protons (-CH_2_), respectively. A doublet at Δ 7.81 ppm for two protons has been assigned to aromatic H-5 and H-8 protons of coumarin nucleus. A pair of multiplet resonating at Δ 7.64–7.68 and Δ 7.20–7.53 have been allocated to H-6, H-7 (coumarin nucleus) (2 protons) and pyridine nucleus (8 protons), respectively. In ^13^C NMR spectral study, the absorption bands resonating at Δ 181.79 and Δ 166.81 have been assigned to *α*, *β*-unsaturated carbonyl and lactone carbonyl groups, respectively (S3 Fig). The spectrum also displayed the presence of three methylene carbons (-CH_2_) resonating at Δ 64.20, 61.35 and 61.34, confirming the condensation of two substrate moieties to form ligand-L. The absorption bands Δ 116.53–157.85 have been assigned to other aromatic ring carbons of coumarin and pyridine nucleus. The mass spectral analysis was found to be in good conformity with the proposed structure.

### Absorption studies of ligand-L with ctDNA and Cu(II)

**Fig 2:**
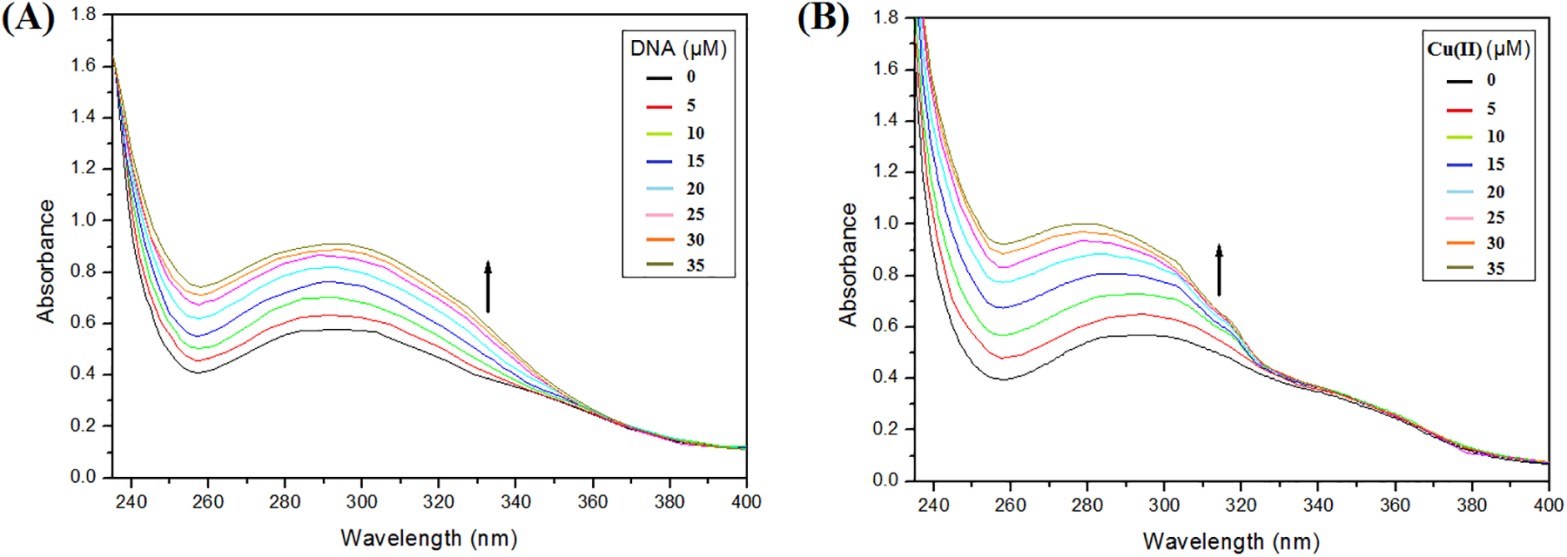
Absorption spectra of ligand-L (5 μM) in the absence and presence of ct-DNA (0–35 μM) and Cu(II) (0–35 μM). (A) Increasing concentration of ct-DNA showed hyperchromic shift suggesting ct-DNA-Ligand-L interaction. (B) Increasing concentration of Cu(II) showed hyperchromic shift suggesting Cu(II)- Ligand-L interaction.

To understand the interaction of ligand-L with ctDNA and Cu(II) ions, UV-Vis spectroscopy was performed. Results showed that ligand-L exhibits maximum absorbance at ~292 nm (Fig 2). Addition of increasing concentration of ctDNA resulted in hyperchromism with no shift in the position of maximum absorption peak (Fig 2A). In general, reports suggest that hyperchromism is due to interaction of molecules outside the DNA helix, whereas hypochromism is suggestive of intercalatory mode of binding between ligand and DNA [57, 58]. Hence, absorbance spectral studies provide an evidence of non-intercalative binding mode of ligand-L with DNA. Further, no clear isosbestic point suggests more than one type of binding between ligand-L and DNA. Absorption studies also indicate that with increasing concentration of Cu(II), hyperchromism with a blue shift was observed suggesting interaction between ligand-L and Cu(II) ions (Fig 2B). Also, absence of any isosbestic point in ligand-L-Cu(II) spectra suggests more than one type of complex formation between ligand-L and Cu(II). These results confirm the binding of ligand-L to ct-DNA and Cu(II) ions.

### Interaction studies via steady state fluorescence

**Fig 3:**
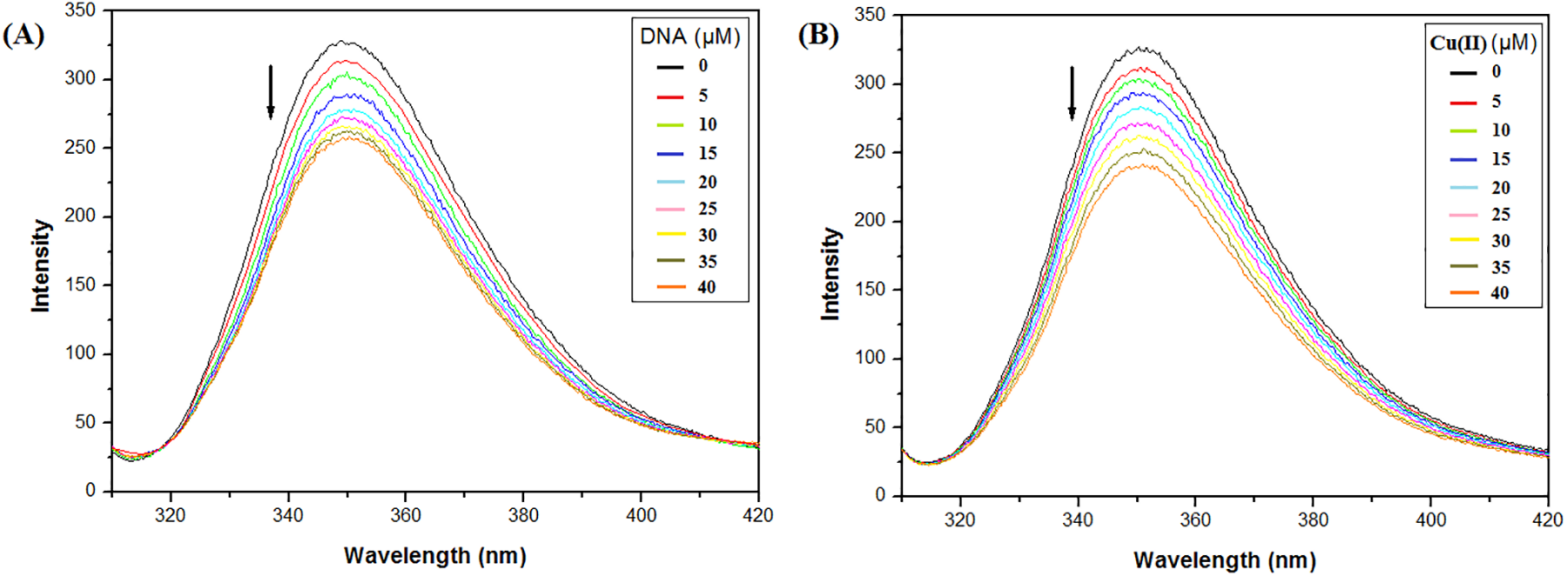
Fluorescence emission spectra of ligand-L (5 μM) in the absence and presence of ct-DNA (0–40 μM) (A) and Cu(II) (0–40 μM) (B). Increasing concentrations of DNA and Cu(II) lead to quenching in fluorescence intensity of ligand-L.

Fluorescence spectroscopy has been widely used to determine the interaction and binding mode of drugs/molecules to DNA due to high sensitivity and accuracy [59]. In the absence of ctDNA or Cu(II) ions, ligand-L exhibited an emission maximum at 350 nm after excitation at 292 nm (Fig 3). On addition of increasing concentrations of DNA, quenching with no shift in emission maxima peak position of ligand-L was observed (Fig 3A). Similarly, addition of increasing concentrations of Cu(II) to ligand-L solution resulted in quenching of fluorescence emission intensity with no significant shift in λ_max_ emission of ligand-L (Fig 3B). This quenching (hypochromism) confirms the interaction of ligand-L with ctDNA and Cu(II) ions.

**Fig 4:**
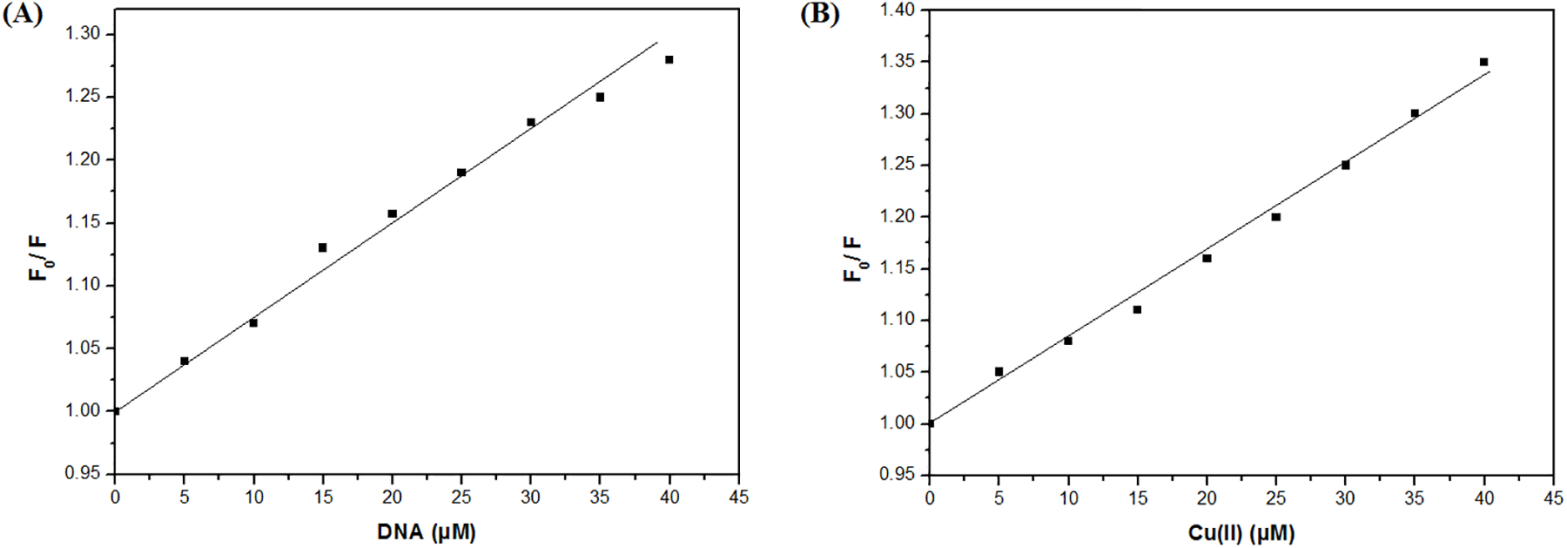
Stern-Volmer plots for interaction of ligand-L with ct-DNA (A) and Cu(II) (B).

To further understand the interaction of ligand-L with DNA and Cu(II) ions, quenching constant (K_sv_) was obtained from Stern-Volmer equation [45, 46]:

$$\frac{F_{0}}{F}=1+K_{sv}\;[Q]$$

where, F_0_ and F are the fluorescence intensities in the absence and presence of ctDNA or Cu(II) ions, respectively and [Q] is the concentration of ctDNA or Cu(II) ions in the solution. K_sv_ was determined by plotting the ratio of fluorescence intensity (F_0_/F) in the absence and presence of ctDNA or Cu(II) ions as a function of increasing concentrations of DNA or Cu(II) ions. K_sv_ values were calculated from the slopes of Fig 4 and found to be (7.41 ± 0.01) × 10^3^ M^-1^ and (8.53 ± 0.01) × 10^3^ M^-1^ for ctDNA and Cu(II) ions, respectively. Ligand-L-DNA complex exhibits a quenching constant value lower than the classical intercalators [60, 61], and therefore indicating non-intercalative mode of binding with DNA.

Later, steady state fluorescence data was also used to calculate binding stoichiometry (n) and binding constant (K) of ligand-L-DNA and ligand-L-Cu(II) complexes using the equation:

$$log\;\left[{(F}_{0}-F\right)/F]=logK+n\;log[Q]$$

where, F_0_ and F are the fluorescence intensities in the absence and presence of DNA or Cu(II) ions, respectively. [Q] is the concentration of DNA or Cu(II) used in the experiments. The values of binding stoichiometry and binding constant were determined by the slope and intercept of the plot log [(F_0_ –F)/F] vs log [Q], respectively. The results so obtained are summarized in Table 1. It was found that both the ligand-L-DNA and ligand-L-Cu(II) systems exhibit an almost identical stoichiometry or binding sites (n value). This result suggests that the molecular population of two different systems contribute equally in molecular interactions in ligand-L complex formation with DNA and Cu(II).

**Table 1:** Parameters obtained using fluorescence studies.

Complex	K_sv_ (×10^3^) (M^-1^)	K (×10^3^) (M^-1^)	n	R^2^
Ligand-L + DNA	7.41 ± 0.01	4.80 ± 0.03	0.958	0.990
Ligand-L + Cu(II)	8.53 ± 0.01	5.23 ± 0.04	0.957	0.984

Thermodynamic parameters were also calculated to determine the nature of binding forces between ligand-L and DNA/Cu(II). There are several kinds of binding forces that exist in interactions between drug and biomolecules such as hydrophobic forces, van der Waals forces, electrostatic interactions and hydrogen bonds [62, 63]. Enthalpy change (ΔH^0^) and entropy change (ΔS^0^) are the main quantities that determine the interaction forces: (1) ΔH^0^ > 0 and ΔS^0^ > 0, hydrophobic forces; (2) ΔH^0^ < 0 and ΔS^0^ < 0, van der Waals force’s and hydrogen bonds; (3) ΔH^0^ << 0 and ΔS^0^ > 0, electrostatic forces. The values of entropy change and enthalpy change were calculated at different temperatures using the van’t Hoff equation:

$$ln\;K=-\frac{{\Delta H}^{0}}{RT}+\frac{{\Delta S}^{0}}{R}$$

where, K represents the binding constant at absolute temperature and R is the gas constant (1.985 × 10^−3^ kcal mol^-1^ K^-1^). The value of ΔH^0^ and ΔS^0^ were obtained from the slope and intercept of the plot ln k vs 1/T, respectively (S4 and S5 Figs). ΔG was determined at three different temperatures (298K, 303 K and 310 K) using the equation: Δ*G* = Δ*H*^0^ − *T*Δ*S*^0^. Results of such calculations at different temperatures for the interaction of ligand-L to DNA/Cu(II) are given in S1 and S2 Tables. As evident from the results, negative ΔH^0^ and ΔS^0^ values indicate that the major interaction forces in ligand-L-DNA complexation are hydrogen bonding and van der Waals forces. Negative ΔH^0^ and positive ΔS^0^ confirm the presence of electrostatic interactions between ligand-L and Cu(II). The negative values of ΔG^0^ confirmed that the interaction of ligand-L to DNA/Cu(II) is spontaneous in nature.

### Competitive displacement assay studies

**Fig 5:**
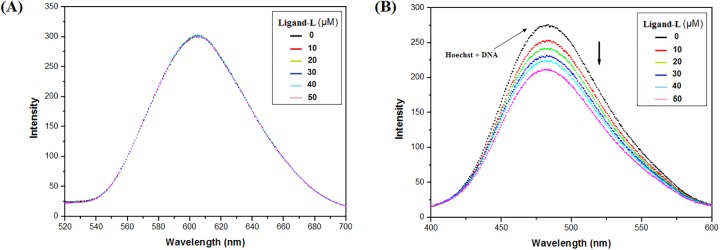
Competitive displacement assays. (A) Fluorescence titration of EtBr-DNA complex with ligand-L. EtBr-DNA complex was excited at 471 nm and emission spectra were recorded from 520–700 nm. No change in fluorescence intensity was recorded on addition of increasing concentration of ligand-L. (B) Fluorescence titration of Hoechst-DNA complex with ligand-L. Hoechst-DNA complex was excited at 343 nm and emission spectra were recorded from 400–600 nm. Fluorescence intensity decreases on addition of increasing concentration of ligand-L.

Competitive binding experiments are extensively used to reveal the binding mode of small molecules to DNA. In order to decipher the binding mode, various DNA binding dyes such as EtBr (intercalator) or Hoechst 33258 (groove binder) are used. This assay is based on the principle that any small molecule/drug that displaces the bound dye from DNA is expected to bind in a similar fashion as bound dye [64–67]. Therefore, changes in the fluorescence of dye-DNA complex upon addition of small molecule/drug help to determine the binding mode of molecule in DNA. As evident from the results of EtBr displacement assay, addition of increasing concentrations of ligand-L did not change the fluorescence intensity of EtBr-DNA complex (Fig 5A). This suggests non-intercalative mode of binding of ligand-L with ctDNA.

To further confirm the non-intercalative mode of binding, Hoechst dye based displacement assay was performed. Hoechst dye holds an ability to bind to the minor groove region of DNA and increases fluorescence intensity on binding to DNA [66, 67]. Results suggest that increase in concentration of ligand-L decreases the fluorescence intensity of Hoechst-DNA system (Fig 5B). This confirms that binding mode of ligand-L is in the groove rather than intercalation.

### Binding studies using ITC method

**Fig 6:**
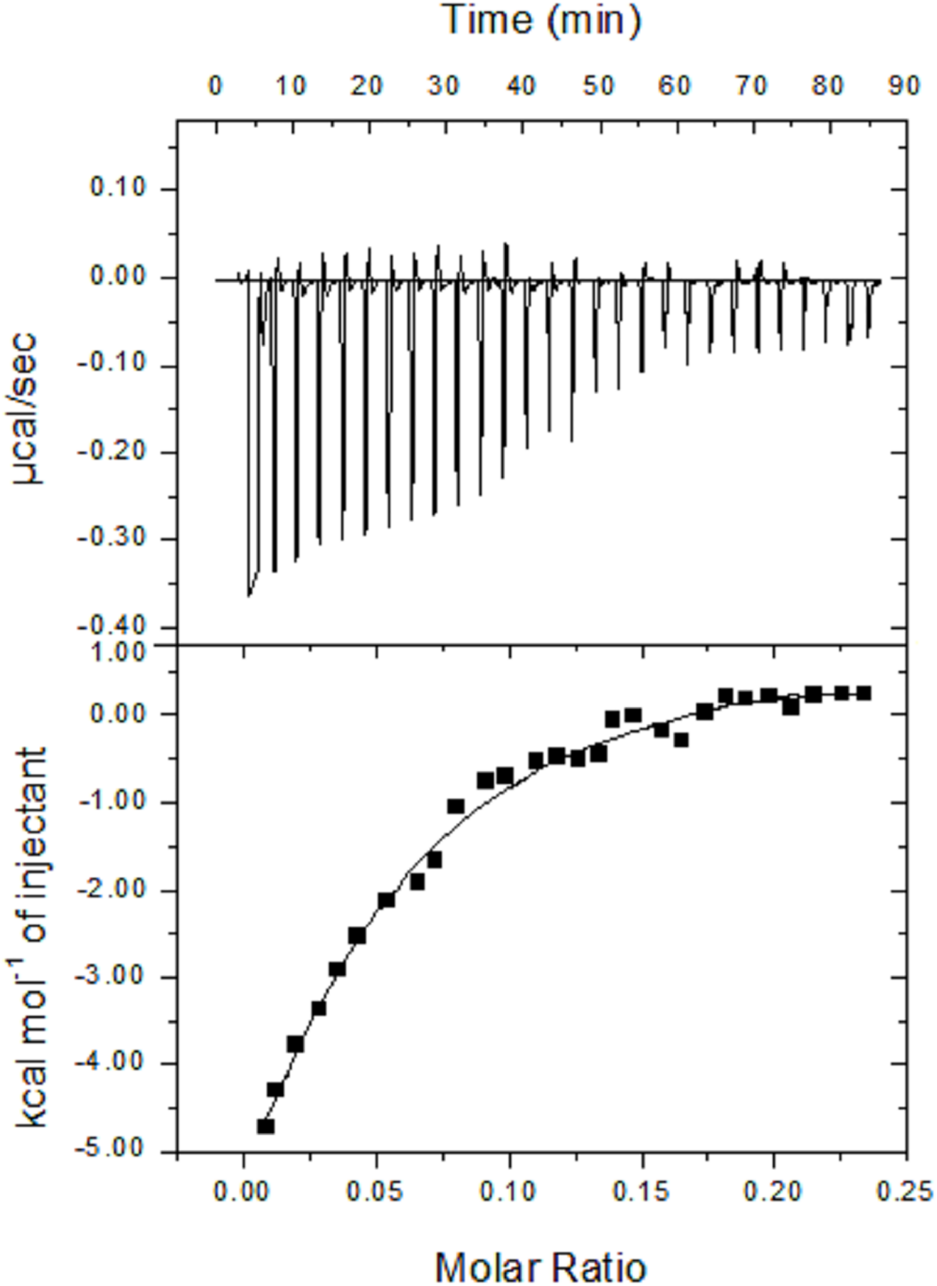
ITC curve (upper panel) and the binding isotherm (lower panel) for ligand-L interaction with ct-DNA at 25°C.

The representative thermodynamic parameters of ligand-L interaction with ctDNA were evaluated using ITC titration method. Table 2 and Fig 6 show the thermodynamic parameters of ligand-L-DNA complex calculated via independent binding model. Ligand-L interaction with DNA resulted in negative value of enthalpy change indicating that the interaction is exothermic in nature. Sequential titrations of ligand-L to DNA solution lead to large negative entropy term (TΔS^0^ = -5.03 × 10^2^ kcal/mol), which suggests that the binding of ligand-L with ctDNA is enthalpy driven. It can also be seen from results in Table 2 that both enthalpy and entropy changes have negative values indicating that the binding of ligand-L with ctDNA is predominately governed by hydrogen bonding and van der Waals interactions. Further, ITC analysis revealed a negative Gibb’s free energy change (∆G^0^) for ligand-L-DNA complex which indicates that the interaction process proceeds spontaneously. It is also important to note that intercalative binding interactions are entropy driven whereas groove binding mode is enthalpy driven [68, 69]. Thermodynamic results obtained from ITC experiment support the competitive binding experiment results (Fig 5), further supporting the non-intercalative binding mode of ligand-L with DNA. Regarding the binding parameters (K_b_ and K_sv_), it should be noted that ITC and fluorescence spectroscopy experiments provide different values (Tables 1 and 2). This difference may be due the fact that ITC measures global or bulk changes in binding and thermodynamic parameters, while fluorescence spectroscopy measures only local changes around the fluorophore [70, 71]. As a result, ITC encounters large molecular interactions among entire molecular population, whereas fluorescence spectroscopy determines only limited or localized molecular interactions in the test sample.

**Table 2:** The binding constant, binding stoichiometry and thermodynamic parameters for the binding of ligand-L to ctDNA determined with ITC at 25°C.

Complex	K_b_ (M^-1^)	n	∆G° (kcal mol^-1^)	∆H° (kcal mol^-1^)	∆S° (kcal mol^-1^ K^-1^)
Ligand-L + DNA	(1.45 ± 0.18) × 10^4^	0.037 ± 0.008	-6.38	(-5.10 ± 0.43) × 10^2^	-1.69

### Circular Dichroism (CD) studies

**Fig 7:**
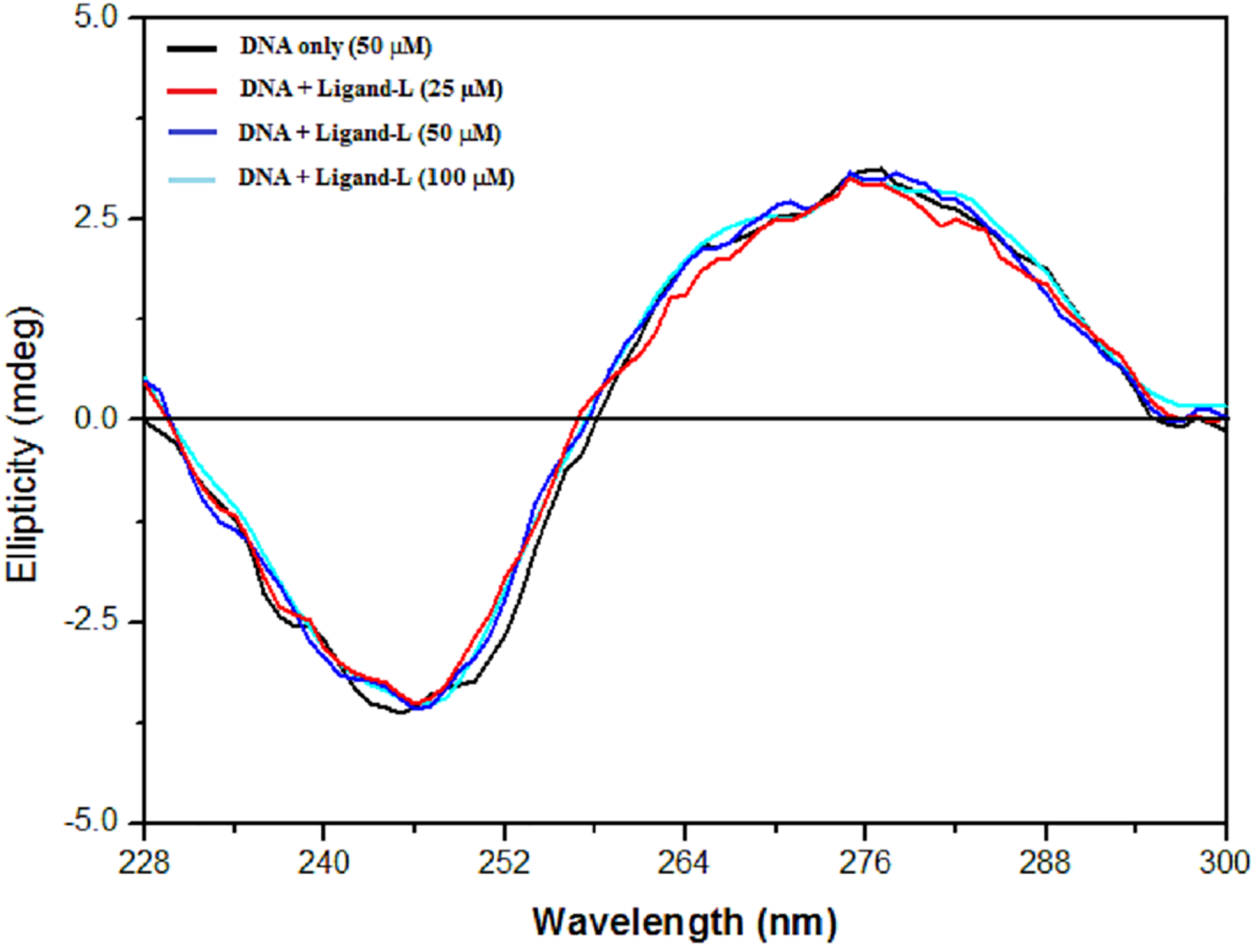
Effect of ligand-L on CD spectra of ctDNA. CD spectra of ctDNA (50 μM) with varying concentrations of ligand-L (0–100 μM).

CD spectroscopy technique is a sensitive method to detect changes in the secondary structure of DNA [72]. Changes in the intrinsic CD spectra of DNA backbone depends on the non-covalent interaction of molecules with DNA [73]. As seen from Fig 7, CD spectrum of DNA alone exhibits a positive peak at ~ 276 nm and a negative peak at ~ 244 nm. This is consistent with CD spectra of double helical DNA in B conformation [74, 75]. The positive band at ~ 276 nm is due to base stacking and negative band at ~ 244 nm corresponds to helicity of right-handed B-form of DNA [76, 77]. Further, it is important to note that both these bands (peaks) are very sensitive to the interaction of small molecules with DNA [77, 78]. Groove binding molecules cause less or no perturbation on the positive and negative bands of CD spectra of ctDNA, whereas intercalating molecules are known to produce significant effect on intensities of both bands [79, 80]. Binding mode of ligand-L with ctDNA was studied using CD spectroscopy. Fig 7 shows that no detectable change in CD spectrum was recorded upon addition of ligand-L to ctDNA solution. These results confirmed the non-intercalative binding mode of ligand-L with ctDNA.

### KI quenching studies

Iodide quenching studies help to determine the binding mode of drug with DNA. Such an assay depends on the fact that when small molecules are intercalated in DNA, iodide ions are repelled by negatively charged phosphate groups of DNA and fluorescence of such molecules remains unaffected in the presence of DNA. However, molecules which are present as groove binders (exposed to external surface) are easily approachable to quenchers even in the presence of DNA [81].

**Fig 8:**
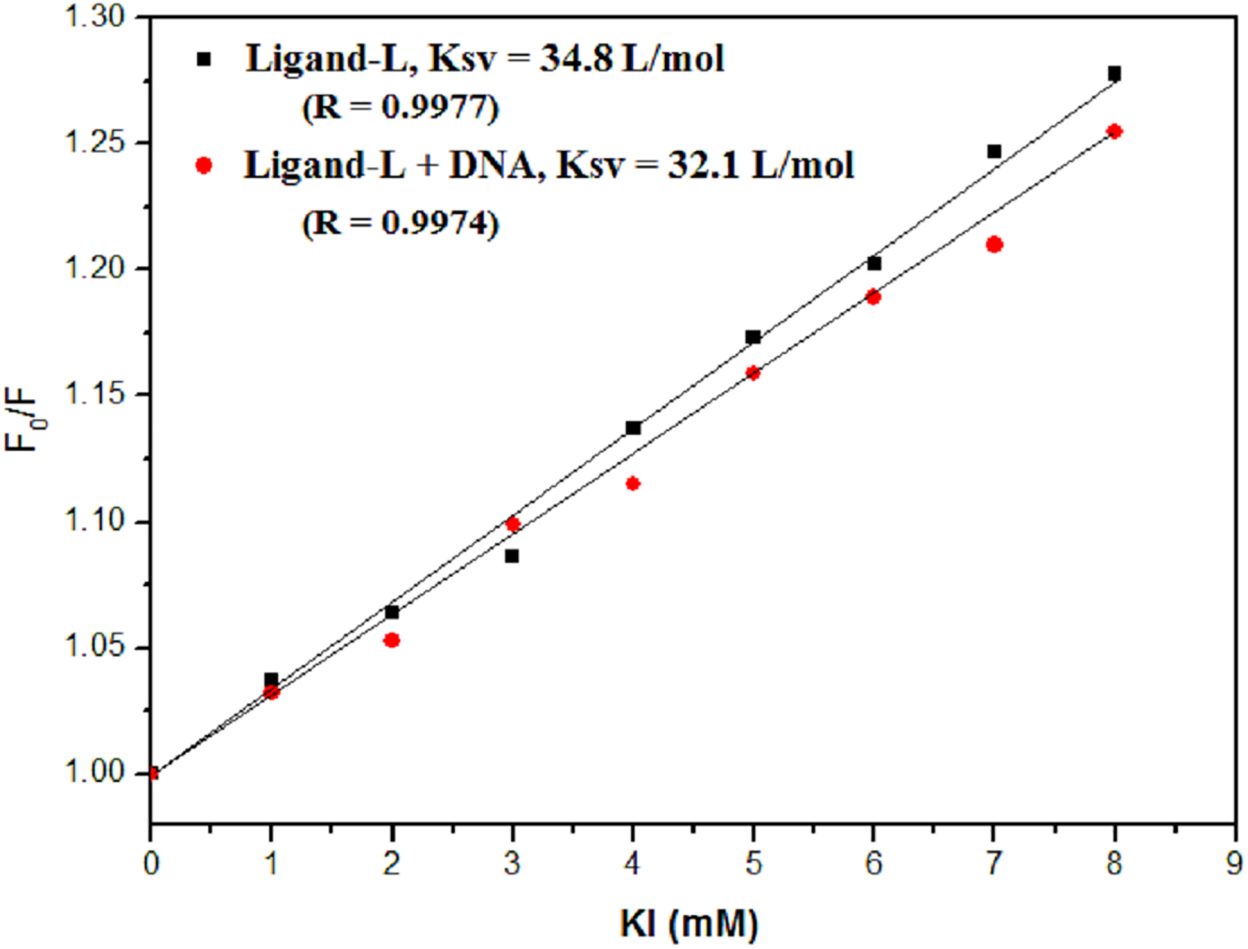
KI quenching experiment. Stern-Volmer plot for fluorescence quenching of ligand-L (30 μM) by KI in the absence and presence of ctDNA (30 μM). Difference in Ksv value (quenching constant) was used to investigate the binding mode of ligand-L with ctDNA. R is the correlation coefficient.

The quenching constant of anionic quenchers in the absence and presence of DNA is calculated via Stern-Volmer equation [45, 46]:

$$\frac{F_{0}}{F}=1+K_{sv}\;[Q]$$

where, F_0_ and F are the fluorescence intensities in the absence and presence of anionic quencher, KI and [Q] is the concentration of KI in the solution. K_sv_ is the quenching constant obtained from the slope of F_0_/F vs [Q] plot. K_sv_ values determine the type of binding of molecules with DNA. Decrease in K_sv_ occurs in intercalation and it remains unchanged when interaction is electrostatic or when molecules bind to the groove. As evident from results, no significant difference in K_sv_ values is observed in the absence and presence of DNA for ligand-L (Fig 8). Therefore, it can be inferred that ligand-L exhibits non-intercalative binding mode with ctDNA.

### Molecular docking studies

**Fig 9:**
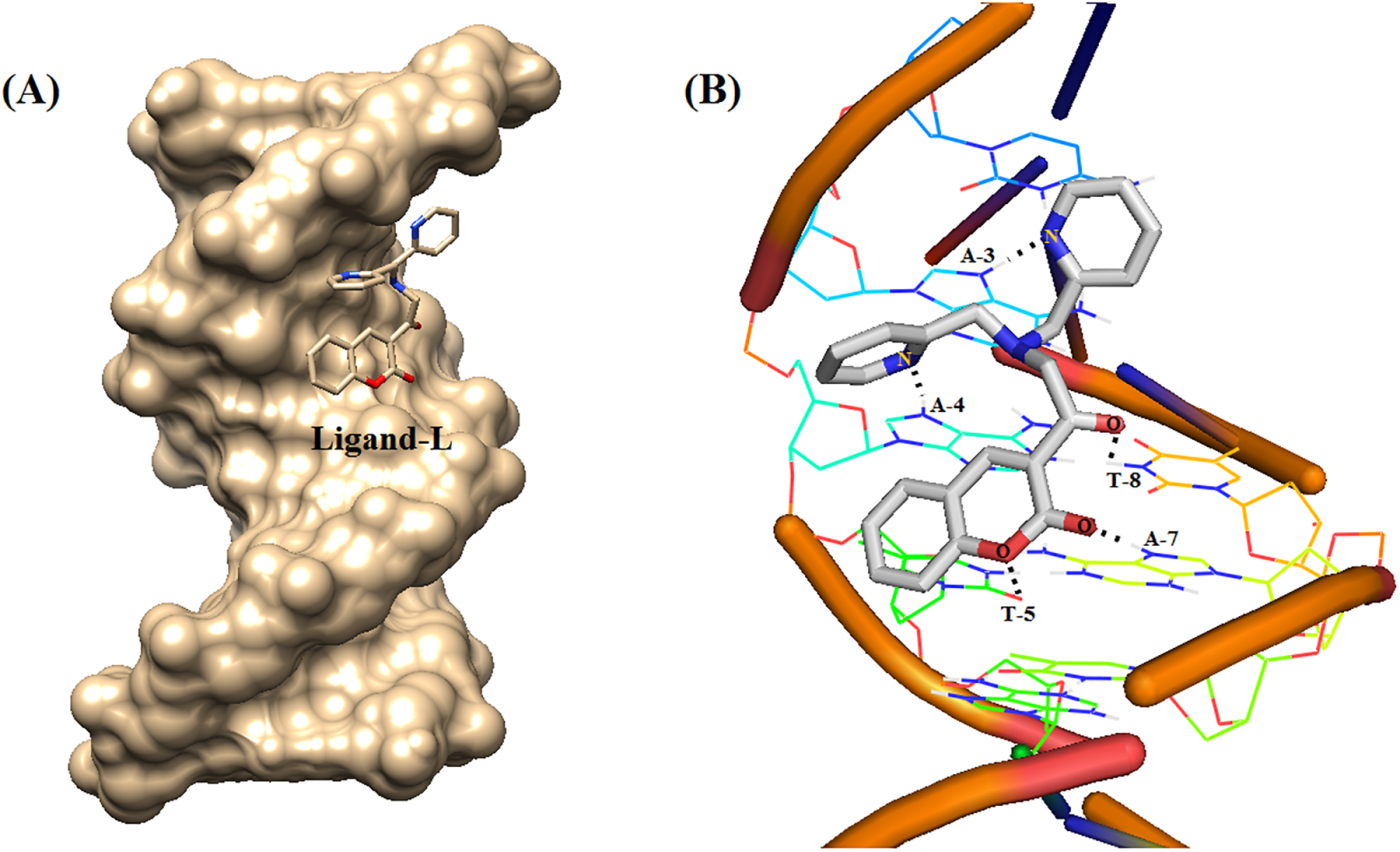
Molecular docked structure of ligand-L complexed with B-DNA. (A) Surface view interaction of ligand-L with B-DNA. (B) Hydrogen bonding interactions (5) of ligand-L with B-DNA (PDB ID: 1BNA).

In order to determine the exact interaction of ligand-L with DNA, docking studies were performed (Table 3 and Fig 9). Surface view interaction of the docked structure in Fig 9A clearly indicates that ligand-L preferentially binds in the groove region of DNA. In total, ligand-L forms 5 hydrogen bonds with adenine (A-3, 4, 7) and thymine (T-5, 8) residues of DNA with binding energy -6.36 kcal/mol and inhibition constant 21.69 μM. Thus, hydrogen bonding plays a major role in the interaction of ligand-L with DNA. More negative binding energy (-6.36 kcal mol^-1^) and lower inhibition constant (21.69 μM) confirm strong binding of ligand-L with B-DNA. Further, higher hydrogen bonding interaction number (5 H-bonds) confirms the greater stability of ligand-L with B-DNA. Finally, it is interesting to note that both the experimental binding free energy (ITC, Table 2) and theoretical binding energy (docking, Table 3) have negative values, indicating that ligand-L-DNA interaction is spontaneous at room temperature. This shows that molecular docking results are in accordance with the results of ITC experiments.

**Table 3:** AutoDock results (binding energy, inhibition constant and no. of hydrogen bonds) of ligand-L in B-DNA.

Ligand	AutoDock binding energy (kcal/mol)	AutoDock inhibition constant (μM)	No. of Hydrogen bonds
**Ligand-L**	-6.36	21.69	5

### Cyclic voltammetric studies of Cu(II)-ligand-L interaction

**Fig 10:**
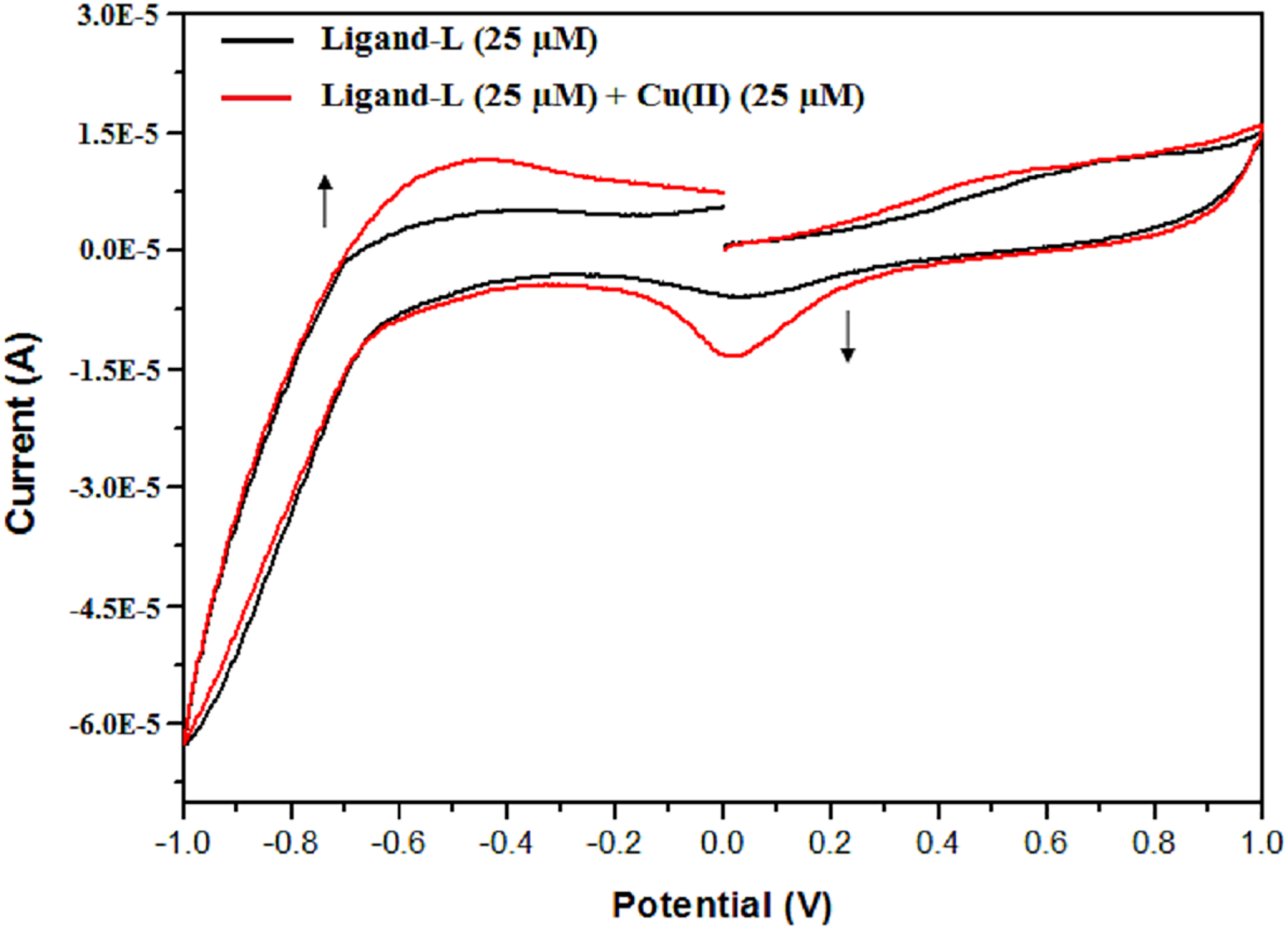
Cyclic voltammogram of ligand-L (25 μM) in absence and presence of Cu(II) (25 μM).

Cyclic voltammetry is an important technique for characterizing the redox properties of metal complexes [82]. The cyclic voltammograms (CV) recorded at a scan rate 150 mV sec^-1^ for ligand-L and Cu(II)-ligand-L solution are shown in Fig 10. CV of ligand-L shows flattened cathodic and anodic peaks. However, addition of Cu(II) ions to ligand-L solution leads to the formation of pronounced cathodic and anodic peaks at $E_{p}^{c}$ = 0.03 V and $E_{p}^{a}$ = -0.45 V, respectively. These two peaks couple together to form a well-defined Cu(II)/Cu(I) qausi-reversible redox couple [83, 84] with E^0^_½_ = 0.48 V (half-wave potential). The separation between the cathodic and anodic peak current (I_pa_/I_pc_ = 0.84) clearly confirms the formation of redox couple Cu(II)/Cu(I) via one electron transfer redox process.

### *In silico* study of ADME/T prediction

For a compound to be orally active (drug-likeness), it should follow Lipinski ‘Rule of five’ which includes the following parameters: (1) Mass < 500; (2) Octanol-water partition coefficient (logP) ≤ 5; (3) Rotatable bonds ≤ 10; (4) Polar surface area ≤ 150 Å^2^; (5) Hydrogen bond acceptors ≤ 10; (6) Hydrogen bond donors ≤ 5. Ligand-L under investigation follows all the parameters under ‘Rule of five’ (no violations) calculated via molinspiration and chemicalize.org servers, and revealed higher tendency of ligand-L towards drug-likeness (S3 Table).

### Ligand-L exerts no toxic effect on RBCs *in vitro*

**Fig 11:**
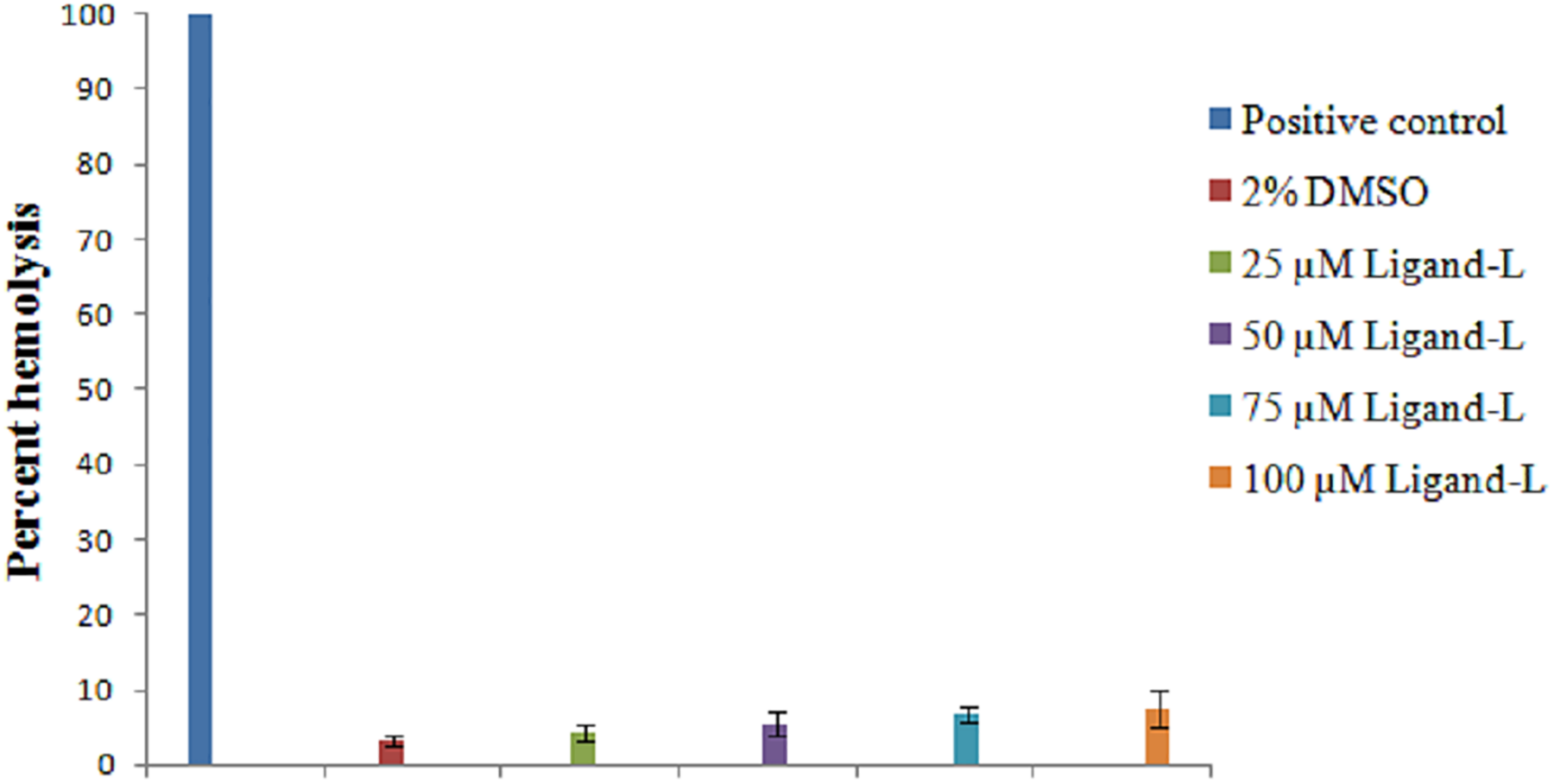
Erythrocyte lysis test. *In vitro* toxicity measured on treatment with vehicle control (2% DMSO) and increasing concentrations of ligand-L (25–100 μM). Values expressed as mean ± SEM of three independent experiments.

Toxicity is the major side effect of synthesized cancer chemotherapeutic agents. Therefore, we investigated the toxic effect of ligand-L by *in vitro* RBC lysis testing (Fig 11). As evident from our results, ligand-L did not induce significant hemolysis and such an effect was also not observed even at high concentrations 100 μM. These results clearly indicate ligand-L is safe, free of any toxicity constraints and exhibits blood compatibility.

### Ligand-L exerts no toxic effect on liver and kidney

**Fig 12:**
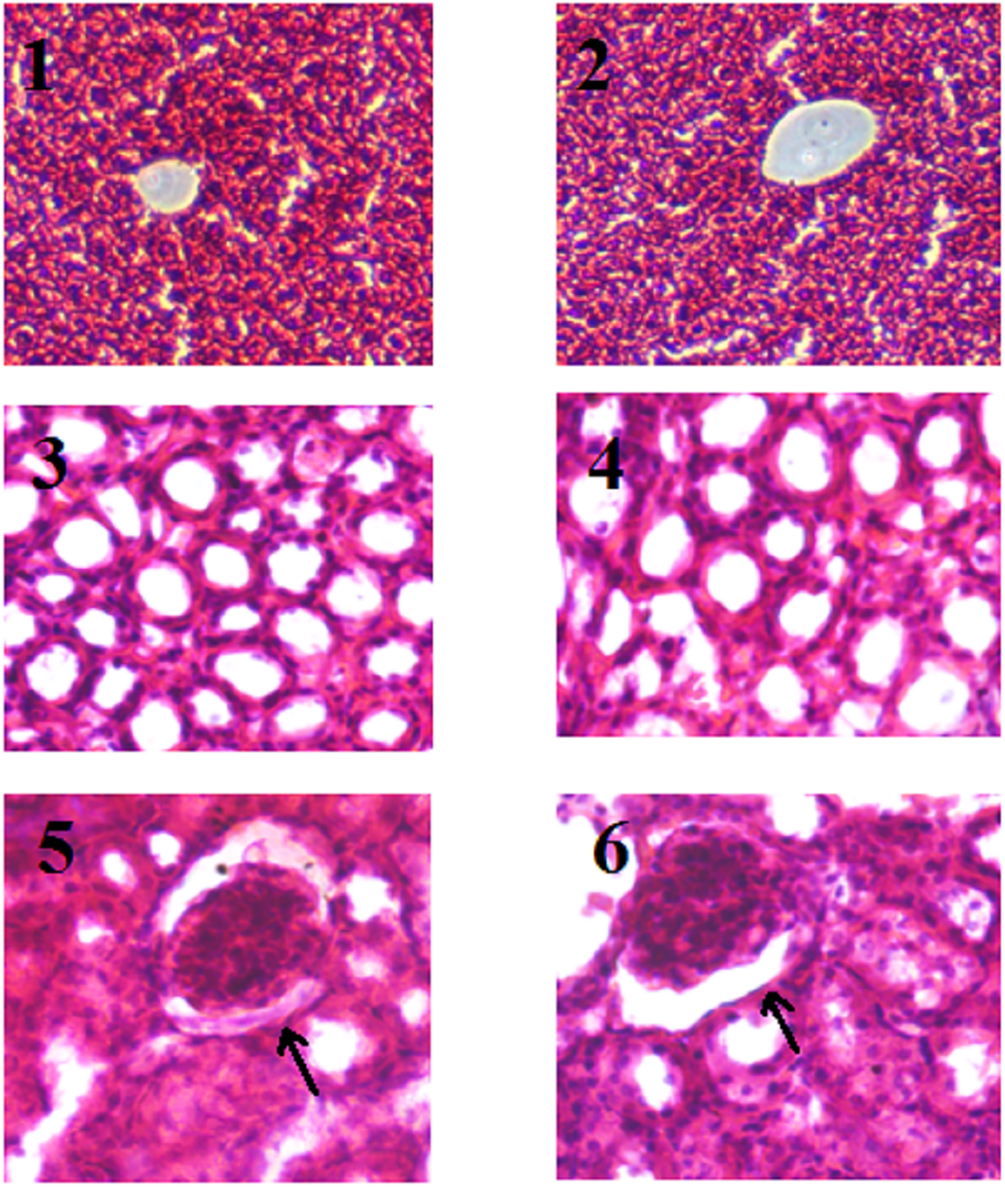
Histological analysis of ligand-L treated groups (H&E staining). Histological sections of liver (first row) and kidney (second and third row). Untreated (control group) liver (Panel 1) and kidney (Panels 3 and 5) of mice. Liver (Panel 2) and kidney (Panels 4 and 6) from ligand-L (250 mg/kg body weight) treated mice. No significant differences in structures were observed of liver and kidney of control and treated groups. Panels 3 and 4 represent tubular region of control and treated kidney respectively, whereas panels 5 and 6 represent glomerulus region of control and treated kidney respectively.

Acute toxicity of ligand-L was evaluated using albino mice. Mice well tolerated the drug and no sign of pharmacotoxicity was observed at 250 mg/kg body weight dose. Liver and renal function test biomarkers in the serum of control and treated-mice are reported in Tables 4 and 5 and no significant difference was observed in control and treated liver/kidney of mice. Further, no obvious histopathological changes were observed in treated liver and kidney structures as compared to normal control group (Fig 12). These results clearly suggest that ligand-L is devoid of hepatotoxicity and nephrotoxicity effects and could be used as anticancer agent.

**Table 4:** Effect of ligand-L treatment on liver function test of mice. Values expressed as mean ± SEM.

Group	Total bilirubin (μmol/L)	Albumin (g/L)	ALP (IU/L)	ALT (IU/L)	AST (IU/L)	GGT (IU/L)
**A: Vehicle**	1.01 ± 0.41	20.55 ± 1.55	40.72 ± 1.27	52.10 ± 2.54	187.15 ± 3.18	3.28 ± 0.28
**B: 250 mg per kg dose**	0.83 ± 0.62	22.32 ± 2.21	43.17 ± 0.73	50.73 ± 3.21	193.2 ± 4.3	3.0 ± 0.11

**Table 5:** Effect of ligand-L treatment on renal function test of mice. Values expressed as mean ± SEM.

Group	Na^+^ (mmol/L)	K^+^ (mmol/L)	Cl^-^ (mmol/L)	Urea (mmol/L)	Creatinine (μmol/L)
**A: Vehicle**	142 ± 1.35	7.7 ± 0.47	111.83 ± 1.55	8.3 ± 2.03	18.23 ± 0.51
**B: 250 mg per kg dose**	145.8 ± 0.34	8.2 ± 0.18	109.1 ± 0.91	9.4 ± 1.11	18.07 ± 0.78

### Cu(II)-ligand-L interaction induces ROS generation

**Fig 13:**
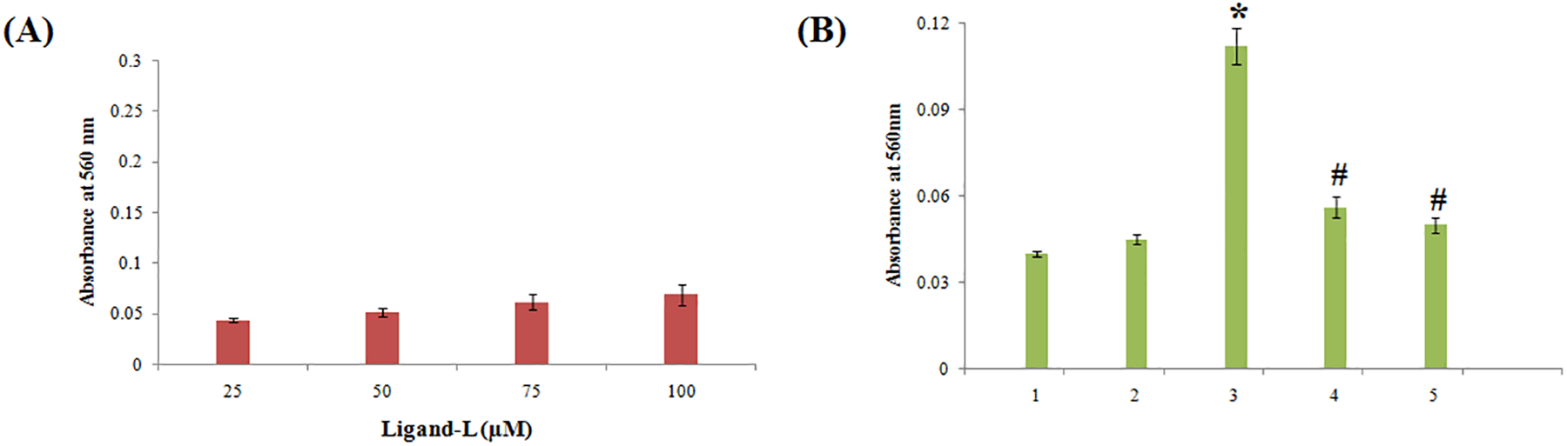
NBT reduction assay. (A) Estimation of superoxide anion generation by increasing concentrations of ligand-L (25–100 μM). (B) Superoxide anion generation by ligand-L in the presence of Cu(II) ions and effect of copper chelator (neocuproine) and superoxide dismutase (ROS scavenger) on ROS generation by ligand-L-Cu(II) system. (1) Ligand-L (25 μM) (2) Cu(II) (25 μM) (3) Ligand-L (25 μM) + Cu(II) (25 μM) (4) Ligand-L (25 μM) + Cu(II) (25 μM) + Neocuproine (50 μM) (5) Ligand-L (25 μM) + Cu(II) (25 μM) + SOD (20 μg/ml). *P < 0.05 with respect to ligand-L (25 μM) set and #P < 0.05 with respect to ligand-L (25 μM) + Cu(II) (25 μM) set.

**Fig 14:**
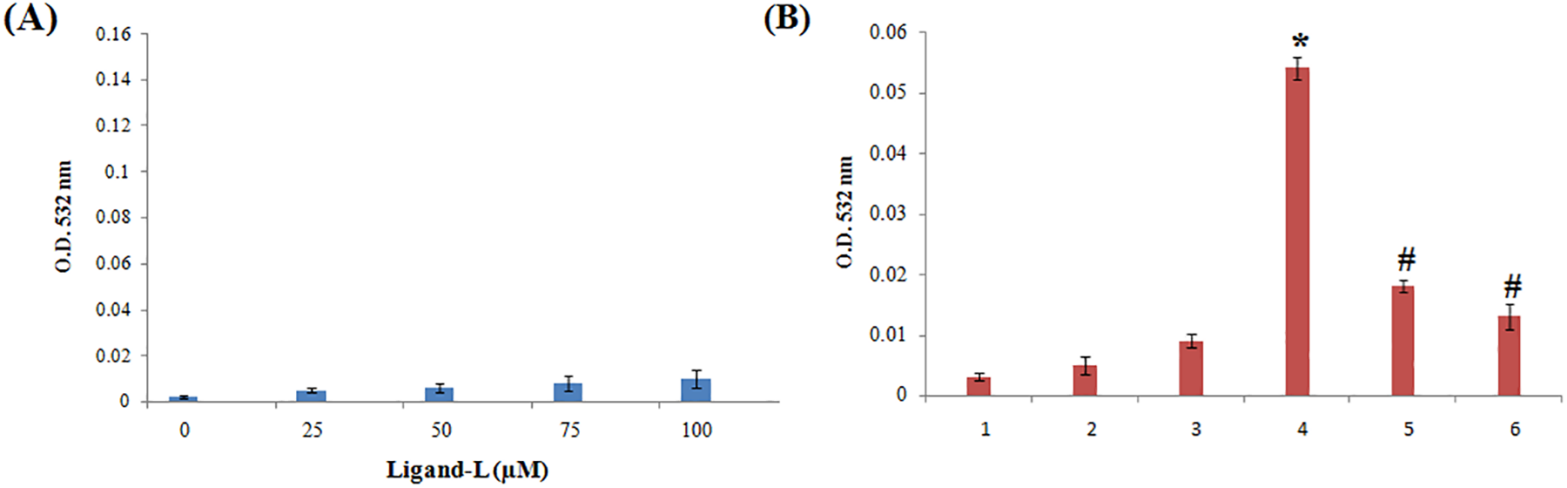
(A) Estimation of hydroxyl radical generation by increasing concentrations of ligand-L (25–100 μM). (B) Hydroxyl radical generation by ligand-L in the presence of Cu(II) ions and effect of copper chelator (neocuproine) and thiourea (ROS scavenger) on ROS generation by ligand-L-Cu(II) system. (1) Control-No treatment (2) Ligand-L (25 μM) (3) Cu(II) (25 μM) (4) Ligand-L (25 μM) + Cu(II) (25 μM) (5) Ligand-L (25 μM) + Cu(II) (25 μM) + Neocuproine (50 μM) (6) Ligand-L (25 μM) + Cu(II) (25 μM) + thiourea (0.2 mM). *P < 0.05 with respect to control and #P < 0.05 with respect to Ligand-L (25 μM) + Cu(II) (25 μM) set.

Increased oxidative stress beyond the threshold level serves as an effective therapeutic strategy to target cancer cells [85]. ROS mainly include superoxide anion (O_2_^.-^) and hydroxyl radical (OH^.^) which are harmful to bio-macromolecules of cells. Superoxide anion and hydroxyl radicals were estimated using NBT assay and ctDNA substrate assay (Figs 13 and 14). As evident from results of Fig 13A, ligand-L alone did not induce significant superoxide anion generation. Addition of Cu(II) ions in ligand-L solution leads to significant superoxide anion generation (Fig 13B). Such superoxide anion generation was quenched by copper chelator, neocuproine (Fig 13B). Further, the presence of SOD (enzymatic ROS scavenger) largely abrogated the superoxide generation by Cu(II)-ligand-L interaction (Fig 13B). Fig 14 demonstrates the results of hydroxyl radical generation by ligand-L in the absence and presence of Cu(II) ions. Ligand-L did not induce significant hydroxyl radical generation (Fig 14A). However, presence of Cu(II) in ligand-L solution causes significant hydroxyl radical production (Fig 14B). Also, hydroxyl radical generation was abrogated on addition of neocuproine and thiourea (Fig 14B). These results confirm that ligand-L causes redox cycling of Cu(II) ions leading to ROS generation.

### Treatment of supercoiled plasmid pBR322 with Cu(II)-ligand-L system

**Fig 15:**
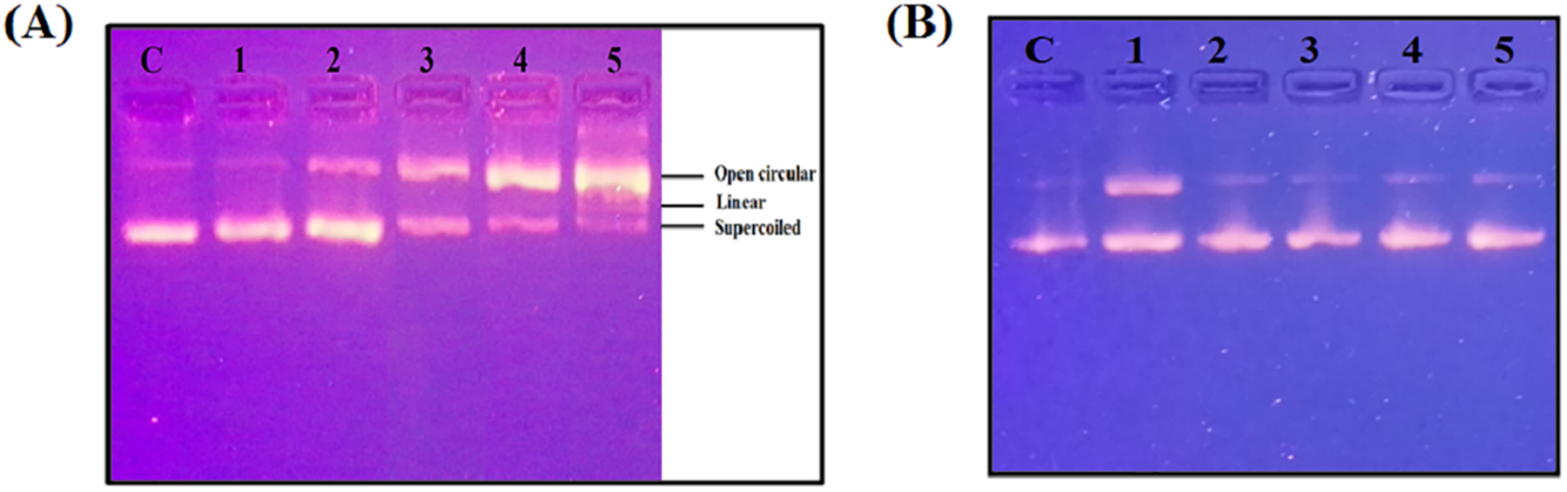
Cu(II)-Ligand-L system induced plasmid DNA damage. (A) Treatment of plasmid pBR322 DNA with ligand-L alone (25 μM) (Lane 1) and Cu(II)-Ligand-L system i.e. Ligand-L (25 μM) + Cu(II) (25 μM) (Lane 2), Ligand-L (25 μM) + Cu(II) (50 μM) (Lane 3), Ligand-L (25 μM) + Cu(II) (75 μM) (Lane 4), Ligand-L (25 μM) + Cu(II) (100 μM) (Lane 5). (B) Effect of copper chelator (neocuproine) and ROS scavengers (thiourea, catalase and SOD) on plasmid pBR322 DNA damage induced by ligand-L-Cu(II) system. (Lane1 1): Ligand-L (25 μM) + Cu(II) (25 μM). (Lane 2): Ligand-L (25 μM) + Cu(II) (25 μM) + Neocuproine (50 μM). (Lane 3): Ligand-L (25 μM) + Cu(II) (25 μM) + thiourea (0.2 mM). (Lane 4): Ligand-L (25 μM) + Cu(II) (25 μM) + catalase (20 μg/ml). (Lane 5): Ligand-L (25 μM) + Cu(II) (25 μM) + SOD (20 μg/ml). Lane ‘C’ depicts the ‘Control’ untreated plasmid DNA.

Cytotoxicity of Cu(II)-ligand-L system was assessed on supercoiled pBR322 plasmid DNA. As seen from the agarose gel pattern, increasing concentrations of ligand-L alone (25–75 μM) and Cu(II) alone (25–75 μM) did not cause any DNA cleavage (S6 Fig). On the other hand, increasing concentration of Cu(II) (25–100 μM) in the presence of 25 μM ligand-L leads to nicking of plasmid from its supercoiled form to open circular topological structure of DNA (Fig 15A). At molar ratio 1:4 of ligand-L:Cu(II) system, the cleavage becomes more pronounced with the appearance of linear form of plasmid DNA (Fig 15B). Further, addition of neocuproine and ROS scavengers abrogated plasmid nicking via ligand-L:Cu(II) system [Ligand-L (25 μM) + Cu(II) (25 μM)] (Fig 15B). These results suggest that ligand-L is capable of DNA damage in the presence of copper via redox cycling of copper ions (Cu(I) acts as intermediate in DNA damage) and ROS generation.

## Discussion

Copper uptake is markedly increased in all types of tumor cells as it is required for tumor growth and angiogenesis [86, 87]. Tumor cells express high levels of CTR1, primary copper transporter which aids in the uptake and accumulation of copper required to maintain internal homeostasis [88]. Redox active copper complexes mediate direct DNA stand scission via ROS generation and such complexes have been screened for anticancer activity *in vitro* and *in vivo* [89–91]. The basic rationale behind targeted cancer therapy using redox active copper chelating agents is to generate high levels of ROS to surpass threshold limit leading to oxidative stress mediated irreversible damage and cancer cell death.

Here, we synthesized 3-(2-{bis[(pyridin-2-yl)methyl]amino}acetyl)-*2H*-chromen-2-one (ligand-L) which binds to DNA and Cu(II). *In vitro* fluorescence quenching study reveals that the binding of ligand-L to DNA/Cu(II) is strong because the binding constant, K (Table 1) is high. Therefore, it is very likely that ligand-L will also bind with DNA/Cu(II) in *in vivo* conditions. Our results of high binding constant (~ 10^3^ M^-1^) are supported by the studies present in literature where it was shown that curcumin (binding constant with DNA: 2.97 × 10^3^ M^-1^) causes DNA damage in cancer cells and also inhibits tumor growth in *in vitro* and *in vivo* xenograft model [92–94].

ROS generation and DNA cleavage assays are suggestive of a role of Cu(II)-ligand-L interaction in ROS generation that causes DNA breakage. Further, experiments using neocuproine (copper chelator) and ROS scavengers have shown inhibition of DNA cleavage and ROS generation by Cu(II)-ligand-L complex, suggesting that ligand-L toxicity requires interaction with copper. Thus, copper ions act as a molecular target of ligand-L in mediating redox cycling that leads to ROS generation (hydroxyl radicals) and selective cytotoxicity against cancer cells.

Ligand-L in the presence of transition metals like copper generates ROS via Fenton-like reaction. Such a quasi reversible Cu(II)/Cu(I) redox couple decomposes hydrogen peroxide to generate hydroxyl radicals, which act as proximal DNA cleaving agent [95].

$$Cu\left(I\right)+H_{2}O_{2}\to Cu\left(II\right)+OH^{.}+OH^{-}$$

We believe that copper-dependent ROS generation by ligand-L is the primary step in inducing oxidative stress and DNA damage. Further, up-regulation of p53 mitochondrial gene, ATM/ATR pathway, down-regulation of anti-apoptotic protein (Bcl-2) and cell cycle related proteins (cyclin, cyclin dependent kinases-CDKs) might be the secondary downstream effects of ROS generation induced by Cu(II)-ligand-L interaction [96]. These effects may be ultimately involved in inducing apoptosis of cancer cells.

Among oxygen radicals generated by Cu(II)-ligand-L system, hydroxyl radicals are the most electrophilic with high reactivity and also possess small diffusion radius. For hydroxyl radicals to cause strand scission, ROS should be generated in close proximity of cellular DNA [97–99]. For the pro-oxidant activity of ligand-L, it is important that ligand-L should be associated with DNA prior to generation of hydroxyl radicals at that site. Thus, ligand-L should bind to DNA where it can interact with chromatin bound Cu(II) to form Cu(II)-DNA-ligand-L ternary complex to mediate ROS generation.

To establish the role of ROS in cancer treatment, it is important to note that tumor cells have higher basal levels of intra-cellular ROS as compared to normal cells [100]. Studies also suggest that cancer cells exhibit up-regulated antioxidant system attributed to evade altered redox status [85]. Thus, pro-oxidant approaches (via anticancer agents) should be implemented to further increase the oxidative stress above the toxicity threshold level where the antioxidant system of these cells is also not sufficient to contain toxic ROS levels, representing an effective mechanism for cancer treatment [85, 101].

In summary, we synthesized, characterized and examined the biological relevance of coumarin nucleus-based DPA derivative (ligand-L). A series of biophysical and docking studies were implemented to predict the binding characteristics and interaction of ligand-L with ctDNA and Cu(II). Results clearly confirmed the formation of ligand-L-DNA and ligand-L-Cu(II) complex. Ligand-L engages in Cu(II) redox cycling to generate ROS that leads to DNA damage (nicking). Thus, ligand-L in all probabilities acts as pro-oxidant in the presence of Cu(II), leading to its cytotoxic action. Since tumor cells have elevated copper than normal cells, this provides a window of opportunity for ligand-L to be used as robust therapeutic agent for selective cytotoxic action against different malignancies with copper as the molecular target. Further studies are in progress in our laboratory to provide a deep insight into the molecular mechanism of anticancer activity of ligand-L in malignant cells. We expect this study to establish ligand-L as a lead molecule to identify or synthesize new anti-cancer drugs with better copper chelating and pro-oxidant properties.

## Supporting information

S1 Fig FTIR spectra of ligand-L. (PDF)

S2 Fig ^1^H NMR spectra of ligand-L. (PDF)

S3 Fig ^13^C NMR spectra of ligand-L. (PDF)

S4 Fig Van’t Hoff plot for the interaction of ligand-L with DNA. (PDF)

S5 Fig Van’t Hoff plot for the interaction of ligand-L with Cu(II). (PDF)

S6 Fig Plasmid nicking assay. Treatment of plasmid pBR322 DNA with increasing concentrations of ligand-L alone (25–100 μM) (Lanes 1–3) and Cu(II) ions alone (25–100 μM) (Lanes 4–6). Lane C represents untreated (control) plasmid. Ligand-L and Cu(II) treatment alone were ineffective in plasmid DNA cleavage.
(PDF)

S1 Table Binding and thermodynamic parameters of the ligand-L-DNA system. (PDF)

S2 Table Binding and thermodynamic parameters of the ligand-L-Cu(II) system. (DOCX)

S3 Table Virtual screening of ligand-L showing drug-likeliness by (A) Molinspiration (B) chemicalize.org servers.
(PDF)
