# Supplementary figures and images for: Synthesis of novel coumarin nucleus-based DPA drug-like molecular entity: In vitro DNA/Cu(II) binding, DNA cleavage and pro-oxidant mechanism for anticancer action
Source: PLoS One. 2017 Aug 1;12(8):e0181783. doi: 10.1371/journal.pone.0181783 (PMC5538679; doi:10.1371/journal.pone.0181783)

**S1 Fig.** FTIR spectra of ligand-L.

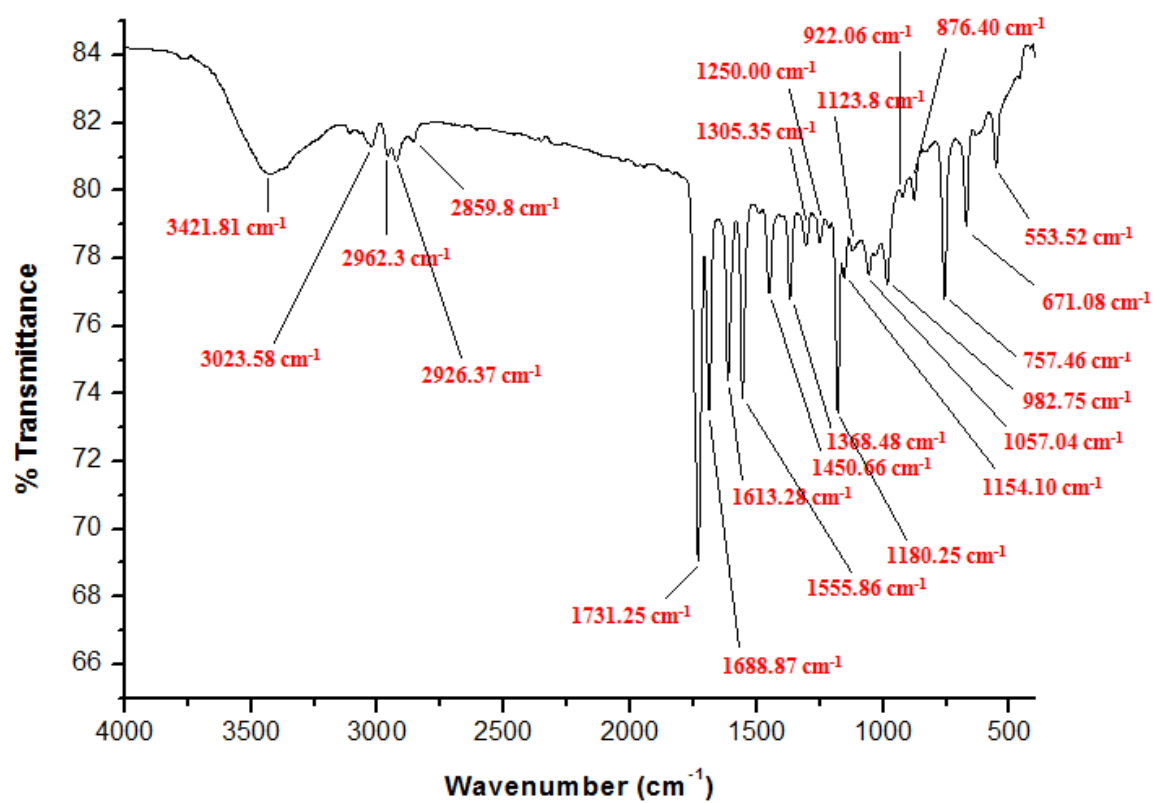

Supplement: S1 Fig — (PDF) [file pone.0181783.s001.PDF]

**S2 Fig.**  $^1\text{H}$  NMR spectra of ligand-L.

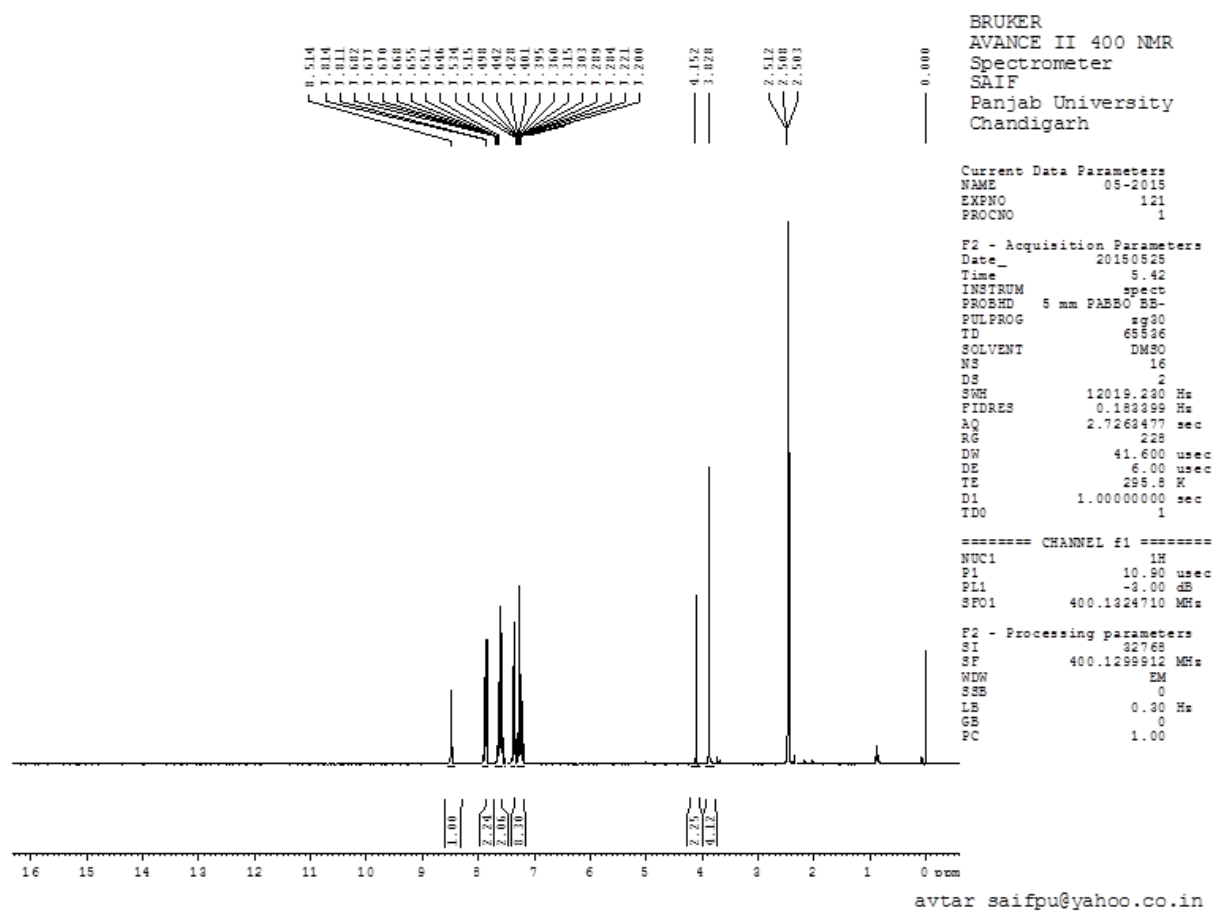

Supplement: S2 Fig — (PDF) [file pone.0181783.s002.PDF]

**S3 Fig.**  $^{13}\text{C}$  NMR spectra of ligand-L.

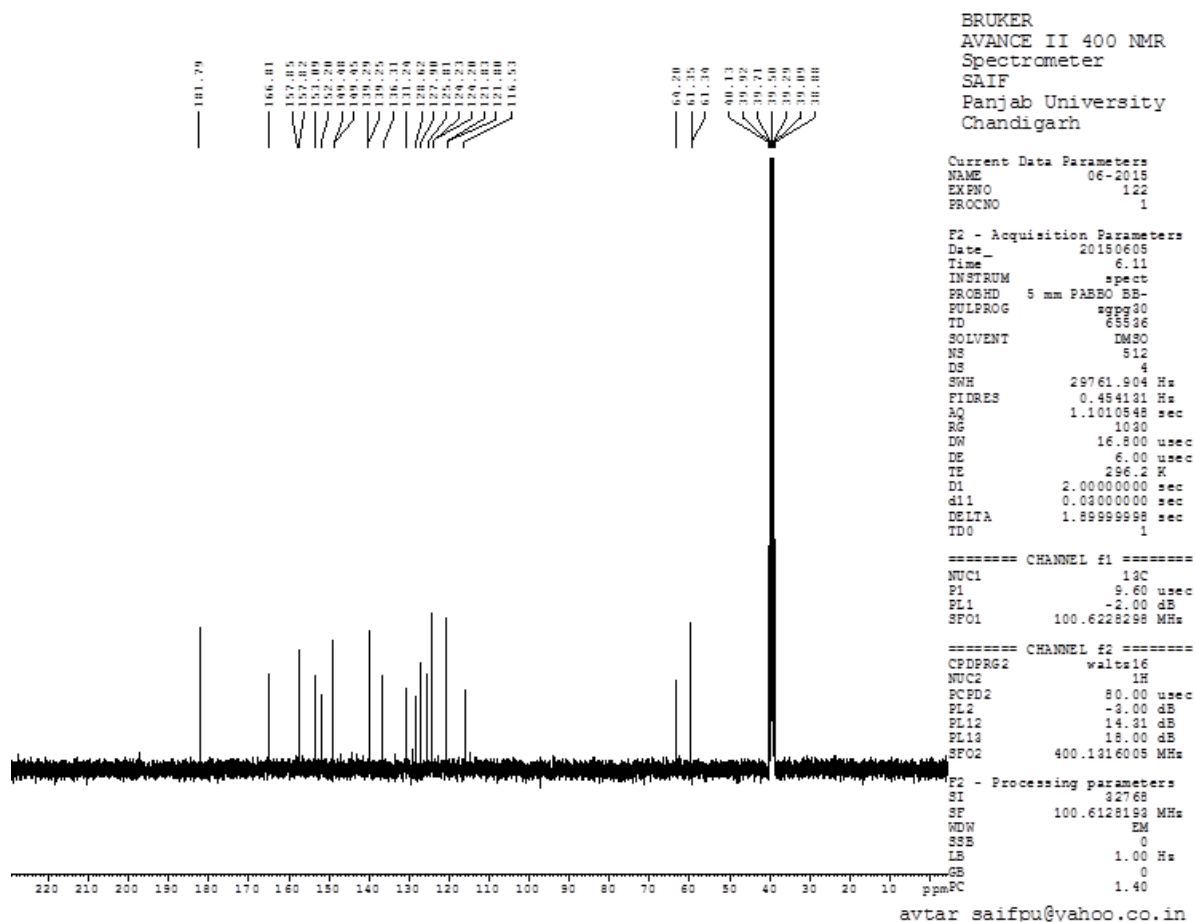

Supplement: S3 Fig — (PDF) [file pone.0181783.s003.PDF]

**S4 Fig.** Van't Hoff plot for the interaction of ligand-L with DNA.

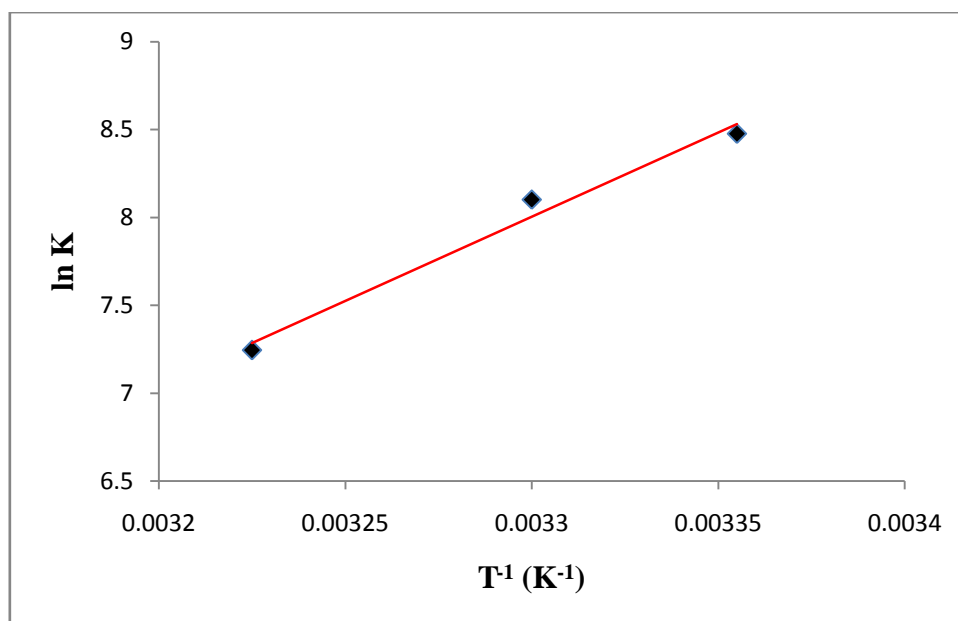

Supplement: S4 Fig — (PDF) [file pone.0181783.s004.PDF]

**S5 Fig.** Van't Hoff plot for the interaction of ligand-L with Cu(II).

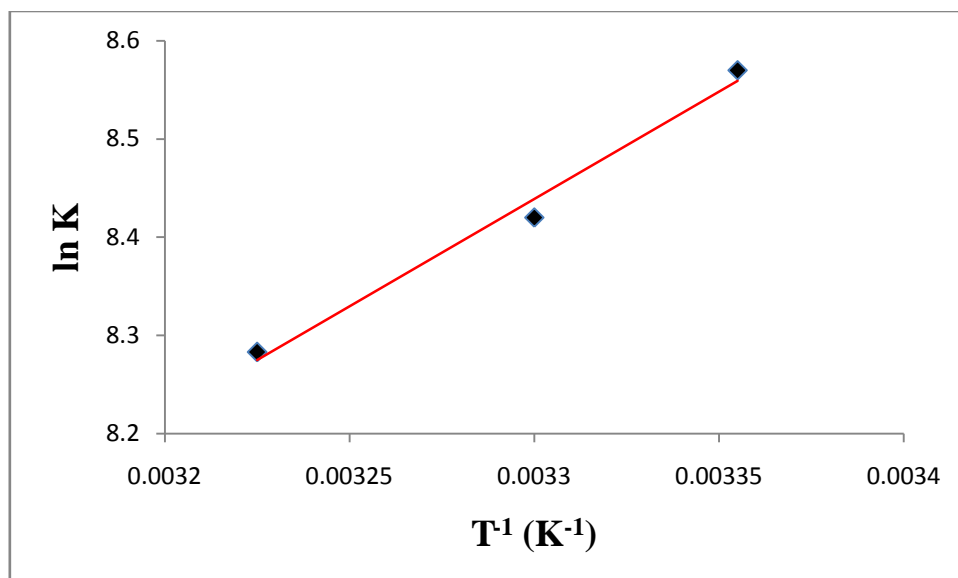

Supplement: S5 Fig — (PDF) [file pone.0181783.s005.PDF]
