# Supplementary material for: Synthesis of novel coumarin nucleus-based DPA drug-like molecular entity: In vitro DNA/Cu(II) binding, DNA cleavage and pro-oxidant mechanism for anticancer action
Source: PLoS One. 2017 Aug 1;12(8):e0181783. doi: 10.1371/journal.pone.0181783 (PMC5538679; doi:10.1371/journal.pone.0181783)

S6 Fig. Plasmid nicking assay. Treatment of plasmid pBR322 DNA with increasing concentrations of ligand-L alone (25-100 μM) (Lanes 1-3) and Cu(II) ions alone (25-100 μM) (Lanes 4-6). Lane C represents untreated (control) plasmid. Ligand-L and Cu(II) treatment alone were ineffective in plasmid DNA cleavage.

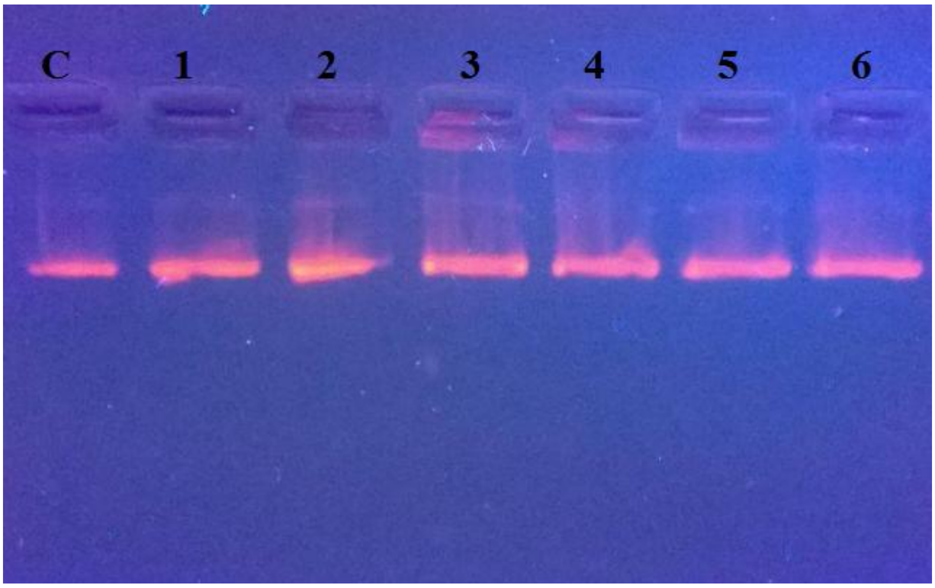

Supplement: S6 Fig — Treatment of plasmid pBR322 DNA with increasing concentrations of ligand-L alone (25–100 μM) (Lanes 1–3) and Cu(II) ions alone (25–100 μM) (Lanes 4–6). Lane C represents untreated (control) plasmid. Ligand-L and Cu(II) treatment alone were ineffective in plasmid DNA cleavage. (PDF) [file pone.0181783.s006.PDF]
