# Supplementary material for: Synthesis of novel coumarin nucleus-based DPA drug-like molecular entity: In vitro DNA/Cu(II) binding, DNA cleavage and pro-oxidant mechanism for anticancer action
Source: PLoS One. 2017 Aug 1;12(8):e0181783. doi: 10.1371/journal.pone.0181783 (PMC5538679; doi:10.1371/journal.pone.0181783)
Supplement: S1 Table — (PDF) [file pone.0181783.s007.PDF]

S1 Table. Binding and thermodynamic parameters of the ligand-L-DNA system.

**Complex**	**Temp (K)**	**K (× 10$^{3)}$ (M^{-1})**	**n**	**ΔH° (kcal mol^{-1})**	**ΔS° (kcal mol^{-1} K^{-1})**	**ΔG° (kcal mol^{-1})**
Ligand-L-DNA	298	4.80 ± 0.03	0.958	-19.05	-0.05	-4.15
	303	3.32 ± 0.02	0.947			-3.90
	310	1.41 ± 0.03	0.935			-3.55
