# Supplementary material for: Synthesis of novel coumarin nucleus-based DPA drug-like molecular entity: In vitro DNA/Cu(II) binding, DNA cleavage and pro-oxidant mechanism for anticancer action
Source: PLoS One. 2017 Aug 1;12(8):e0181783. doi: 10.1371/journal.pone.0181783 (PMC5538679; doi:10.1371/journal.pone.0181783)
Supplement: S2 Table — (DOCX) [file pone.0181783.s008.docx]

**S2 Table.** Binding and thermodynamic parameters of the ligand-L-Cu(II) system.

| **Complex** | **Temp (K)** | **K ( × 10^3)^**  **(M^-1^)** | **n** | **∆H˚**  **(kcal mol^-1^)** | **∆S˚**  **(kcal mol^-1^ K^-1^)** | **∆G˚**  **(kcal mol^-1^)** |
| --- | --- | --- | --- | --- | --- | --- |
| Ligand-L-Cu(II) | 298 | 5.23 ± 0.04 | 0.957 | -4.31 | 0.003 | -3.42 |
|  | 303 | 4.41 ± 0.03 | 0.950 |  |  | -3.40 |
|  | 310 | 3.90 ± 0.03 | 0.952 |  |  | -3.38 |
