# Supplementary material for: Synthesis of novel coumarin nucleus-based DPA drug-like molecular entity: In vitro DNA/Cu(II) binding, DNA cleavage and pro-oxidant mechanism for anticancer action
Source: PLoS One. 2017 Aug 1;12(8):e0181783. doi: 10.1371/journal.pone.0181783 (PMC5538679; doi:10.1371/journal.pone.0181783)
Supplement: S3 Table — Virtual screening of ligand-L showing drug-likeliness by (A) Molinspiration (B) chemicalize.org servers. (PDF) [file pone.0181783.s009.PDF]

S3 Table. Virtual screening of ligand-L showing drug-likeliness by (A) Molinspiration (B) chemicalize.org servers.

**(A) Molinspiration property engine v2014.11**	
miLogP	1.95
TPSA	76.30
natoms	29
MW	385.42
nON	6
nOHNH	0
nviolations	0
nrotb	7

**(B) Chemicalize.org**	
logP	2.77
Polar surface area	72.39
Mass	385.423
No. of H-bond acceptors	5
No. of H-bond donors	0
Molar refractivity	108.47
Rotatable bond count	7
Heavy atom count	29
Lipinski's rule of five	Yes
Bioavailability	Yes
